# Human papillomavirus self-sampling in Asia: a systematic review

**DOI:** 10.3389/fmicb.2025.1540609

**Published:** 2025-03-14

**Authors:** Xuechao Ji, Menglin Hao, Yixiao Wang, Wenzhi Kong, Zangyu Pan, Qi Sun, Jinwei Miao

**Affiliations:** ^1^Department of Gynecologic Oncology, Beijing Obstetrics and Gynecology Hospital, Beijing Maternal and Child Health Care Hospital, Capital Medical University, Beijing, China; ^2^Laboratory for Clinical Medicine, Capital Medical University, Beijing, China

**Keywords:** human papillomavirus, clinician sampling, self-sampling, Asia, cervical cancer, screening

## Abstract

**Background:**

Human papillomavirus (HPV) self-sampling may be an accurate and effective alternative sampling method to conventional cervical cancer screening methods. This systematic review compares the accuracy and acceptance of self-sampling to clinician sampling for HPV testing in Asia.

**Methods:**

The PubMed, Cochrane Library, Cumulative Index to Nursing and Allied Health, and Web of Science databases were searched for publications published from the establishment of the database to 2023. The risk of bias was assessed using the QUADAS-2 tool for studies included in this review. All studies evaluating the accuracy and acceptance of HPV self-sampling, and agreement of self- and clinician-collected samples in Asia were included. The accuracy of each study was demonstrated through the sensitivity and specificity in diagnosing cervical intraepithelial neoplasia or cancer, as well as the detection rate of HPV. The agreement between the two sampling methods was assessed based on the detection outcomes of HPV. Acceptance was indicated by women’s preferences for HPV self-sampling.

**Results:**

Sixty-seven studies including 117,279 adult, female participants were included in this review. The type of HPV screening, other intervention components, study design, sample size, follow-up period, analysis method, numerical outcomes, results, and limitations were extracted from each study. The sensitivity and specificity of HPV self-sampling in detecting cervical intraepithelial neoplasia were higher than 80% and 70%, consistent with the results of HPV clinician sampling. The consistency between self-sampling and clinician-sampling was high in most studies, and the kappa value was more than 0.7. Women had high acceptance of self-sampling but expressed some concerns.

**Conclusion:**

Self-sampling for HPV testing can significantly improve cervical cancer screening coverage, especially in areas with limited medical resources or reluctance to accept physician sampling. In most studies, the accuracy and acceptance of HPV self-sampling was comparable to clinician sampling. However, the diagnostic criteria and HPV detection methods still need to be adjusted due to the low sensitivity of HPV self-sampling in some studies in China and India. Targeted health education should be carried out to improve the acceptance of HPV self-sampling in women.

**Systematic review registration:**

https://inplasy.com/?s=INPLASY202520107, INPLASY202520107.

## Introduction

Cervical cancer is the fourth most common cancer in women, leading to approximately 661,021 cases and 348,189 deaths in 2022 (Bray et al., 2024). Most cervical cancers develop due to persistent high-risk human papillomavirus (HR-HPV) infections (Schiffman et al., 2011). Although vaccines that protect against infections and diseases associated with specific types of HPV exist, many women in low- and middle-income countries do not have access to HPV immunization and die of this preventable cancer (Gallagher et al., 2018). Secondary prevention measures include the early detection and treatment of precancerous lesions (Arbyn et al., 2012). Population-based cervical cancer screening via Papanicolaou testing every three to 4 years has successfully reduced the incidence and mortality of cervical cancer (Bouvard et al., 2021). In organized screening programs, most new cases of cervical cancer are detected in women who have never been screened or are under-screened (Spence et al., 2007). Cervical cancer screening programs, including cervical cytology (Pap smear), visual inspection with acetic acid (VIA), and HPV testing, must be applied to reduce the occurrence of cervical cancer.

Currently, national screening programs for cervical cancer are widely provided in Asian countries including China, India, Japan, and Thailand (Aoki et al., 2020). However, the uptake rates of these programs remain low, indicating that personal barriers hamper the participation of female patients (Chorley et al., 2017; Cremer et al., 2021). It has been hypothesized that offering HR-HPV self-sampling may increase the participation rate compared to clinician sampling (Arbyn et al., 2018; Harding-Esch et al., 2017; Racey et al., 2013; Snijders et al., 2013; Verdoodt et al., 2015). HPV self-sampling may be a more acceptable option for patients in Asia who have never been screened or who are under-screened for cervical cancer. While there have been several systematic reviews on HPV self-sampling globally, there is a notable gap in the literature regarding studies focused specifically on Asian populations. Existing reviews have primarily addressed global or African cohorts, and their findings may not be fully applicable to Asian patients due to differences in cultural, economic, and healthcare factors (Sy et al., 2022). Notably, we have found only one study that has systematically reviewed HPV self-sampling outcomes within India (Hariprasad et al., 2023), but this study did not provide a comprehensive analysis of HPV self-sampling across diverse Asian countries. To our knowledge, no systematic review has reported the sensitivity, specificity, and acceptance of HPV self-sampling in Asia. This systematic review examined the accuracy, agreement, and acceptability of self-sampling for HPV DNA testing in Asian countries.

## Methods

This systematic review was registered with INPLASY (INPLASY202520107, doi: 10.37766/inplasy2025.2.0107), and was conducted in accordance with the Preferred Reporting Items for Systematic Reviews and Meta-Analyses (PRISMA) guidelines (the PRISMA checklist is supplied in Supplementary Table 1) (Page et al., 2021). No funding agency played any role in the study design, data collection, data analysis, data interpretation, or report writing. The review protocol was not registered prospectively.

### Inclusion and exclusion criteria

Articles were included in the review if they included participants who underwent cervicovaginal self-sampling for HPV DNA testing; measured the accuracy, concordance, and acceptability of cervicovaginal self-sampling and clinician sampling for HPV; focused on Asian patients; were conducted in Asian countries and were in English. The included studies were randomized controlled trials, prospective cohort studies, cross-sectional studies, comparative studies, and other non-randomized controlled trials. Studies that did not use vaginal or cervical specimens for examination were excluded from the review. Studies that focused on non-Asian populations, or did not report relevant outcomes related to the accuracy of self-sampling, concordance with clinician-collected samples, or women’s acceptance of self-sampling, were excluded.

### Search strategy

The PubMed, Cochrane Library, Cumulative Index to Nursing and Allied Health Library (CINHAL), and Web of Science databases were searched for studies reported from the establishment of the database to 31 October 2022. A final update of the search was completed before the final extraction and synthesis of the results on 23 February 2023. The reference lists of the included articles were also screened to identify publications that met the eligibility criteria. Database-specific Boolean operators (AND, OR, NOT) and truncation symbols (* and “ “) were used.

The following search terms were used to identify eligible studies:

1. Cervical dysplasia OR cervical intraepithelial neoplasia OR cervix neoplasms OR papillomavirus OR papillomavirus, human OR human papillomavirus OR papillomavirus, infections

AND

2. Self-collected OR self-test OR self-obtained OR self-sampling

AND

3. Asia OR Asian OR Afghanistan OR Armenia OR Azerbaijan OR Bahrain OR Bangladesh OR Bhutan OR Brunei OR Cambodia OR China OR Cyprus OR Georgia OR India OR Indonesia OR Iran OR Iraq OR Israel OR Japan OR Jordan OR Kazakhstan OR Korea, North OR Korea, South OR Kuwait OR Kyrgyzstan OR Laos OR Lebanon OR Malaysia OR Maldives OR Mongolia OR Myanmar OR Nepal OR Oman OR Pakistan OR Palestine OR Philippines OR Qatar OR Saudi Arabia OR Singapore OR Sri Lanka OR Syria OR Tajikistan OR Thailand OR Timor-Leste OR Turkmenistan OR Turkey OR United Arab Emirates OR Uzbekistan OR Vietnam OR Yemen.

The more detailed search strategies of each database were shown in Supplementary Table 2.

### Data collection and analysis

Descriptive data were extracted independently by two authors, and a third reviewer was consulted to resolve any differences in data collection. The citation, objectives, location, population characteristics, description of the type of HPV screening, description of any additional intervention components, study design, sample size, numerical outcomes, results, and limitations were extracted from each included study.

After finalizing the data extraction, two authors reviewed the data and the full texts to accurately classify HPV self-sampling.

The reported data regarding screening accuracy, participation, attendance, response, and compliance were combined to determine the cervical cancer screening outcomes. Conventional cytology (Pap smears), VIA, or colposcopy data were also gathered. When more than one control group was reported, the intervention group was compared to the least intensive sampling strategy group.

Two independent reviewers evaluated the risk of bias for all included studies by using the Quality Assessment Tool for Diagnostic Accuracy Studies-2 (QUADAS-2).

Heterogeneity was assessed using Cochran’s Q test and the I^2^ statistic. Begg’s rank correlation test was performed to further assess publication bias. A funnel plot was used to visualize publication bias.

Human papillomavirus self-sampling was defined as the process in which women insert a self-sampler into their vagina to collect isolated cells. In contrast, HPV clinician sampling involved clinicians or healthcare workers inserting a vaginal speculum into the woman’s vagina to obtain a cervical smear using a sampler.

The diagnostic test sensitivity and specificity were based on colposcopy-confirmed cases of high-grade squamous intraepithelial lesion (HSIL), previously called cervical intraepithelial neoplasia 2+ (CIN2+) or CIN3+, and detection of cervical cancer and HPV infection. The sensitivity was defined as the number of identified cases of HSIL and cervical cancer (positive for both HPV and colposcopy) divided by the total number of colposcopy-confirmed cases. Specificity was defined as the number of cases without HSIL or cervical cancer (negative on both HPV and colposcopy) divided by the total number of colposcopy-negative cases. The HPV detection rate was defined as the HPV-positive cases divided by the total number of women enrolled. Agreement was defined as the concordance between self-sampled HPV tests and clinician-sampled HPV tests (the percentage of agreement with positive test results and the percentage of agreement with negative test results). Acceptability was defined as the percentage of women willing to participate in the HPV test and their preference between HPV self-sampling and clinician sampling.

## Results

### Selection of relevant studies

**FIGURE 1:**
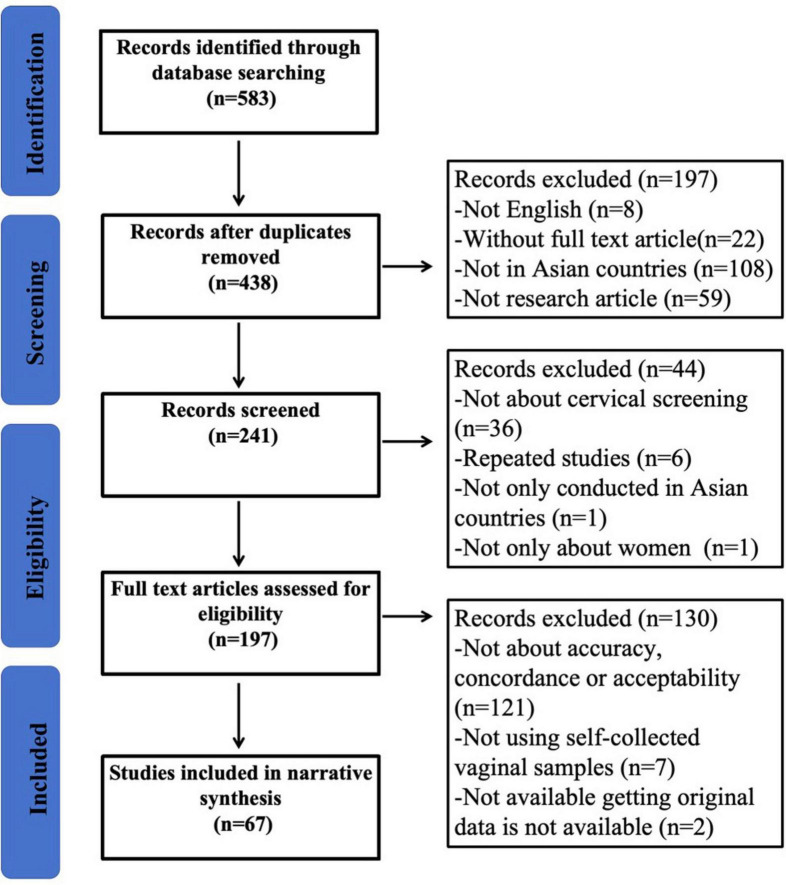
Study selection flow-diagram based on PRISMA guidelines. HPV, human papillomavirus.

A total of 573 articles were retrieved, comprising 124 studies from PubMed, 215 from Web of Science, 26 from the Cochrane Library, and 208 from CINAHL, including 135 duplicate titles. Therefore, 438 articles were screened against the eligibility criteria. Following the exclusion of 241 articles based on their titles and abstracts, the full texts of 195 articles were read, and 67 studies were ultimately included in the systematic review (Figure 1).

### Study characteristics

Most of the included studies were cross-sectional studies (*n* = 62). The remaining studies were randomized controlled trials (*n* = 1), prospective cohort studies (*n* = 1), prospective population-based studies (*n* = 1), and prospective randomized crossover studies (*n* = 2) (Supplementary Table 3).

The patient populations of the included studies were women in China (28 studies) (Belinson et al., 2001; Belinson et al., 2003; Belinson et al., 2010; Belinson et al., 2012; Chang et al., 2002; Chen S. et al., 2014; Chen W. et al., 2014; Chen K. et al., 2016; Chen Q. et al., 2016; Chou et al., 2016; Du et al., 2021; Goldstein et al., 2020; Guan et al., 2012; Guan et al., 2013; He and He, 2020; Li et al., 2022; Ngu et al., 2022; Qiao et al., 2008; Qin et al., 2016; Tisci et al., 2003; Twu et al., 2011; Wang et al., 2014; Wang et al., 2017; Wong et al., 2016; Wong et al., 2018; Wong et al., 2020; Zhang et al., 2020; Zhao et al., 2013), Thailand (nine studies) (Gottschlich et al., 2019; Kittisiam et al., 2016; Nilyanimit, 2014; Nutthachote et al., 2019; Oranratanaphan et al., 2014; Phoolcharoen et al., 2018a; Phoolcharoen et al., 2018b; Ploysawang et al., 2023; Trope et al., 2013), Japan (seven studies) (Aiko et al., 2017; Hanley et al., 2016; Onuma et al., 2020; Ozawa et al., 2023; Satake et al., 2020; Terada et al., 2022; Yoshida et al., 2011), Malaysia (seven studies) (Abdullah et al., 2018; Ahmad et al., 2021; Khoo et al., 2021; Latiff et al., 2015a; Latiff et al., 2015b; Ma’som et al., 2016; Tan et al., 2021), India (six studies) (Anand et al., 2022; Asthana and Labani, 2015; Bhatla et al., 2009; Kuriakose et al., 2020; Madhivanan et al., 2021; Sowjanya et al., 2009), Korea (three studies) (Cho et al., 2019; Seo et al., 2006; Shin et al., 2019), Nepal (two studies) (Johnson et al., 2014; Shrestha et al., 2021), Singapore (Lim et al., 2022), Mongolia (Tsedenbal et al., 2022), Cambodia (Thay et al., 2019), Vietnam (Hanh, 2006), and Brunei (Chaw et al., 2022).

A total of 19 studies evaluated the sensitivity and specificity of clinician-collected and self-collected HPV testing for diagnosing CIN. A total of 35 studies reported the detection rates of HPV using both self-sampling and clinician sampling methods. A total of 29 studies examined concordance between clinician-collected and self-collected HPV testing. A total of 33 studies assessed women’s acceptance and preference rates for HPV self-sampling.

### HPV detection methods of included studies

A total of 13 HPV detection methods are discussed in this review, including seven WHO-approved testing methods: HC2 (Qiagen, Germantown, MD, United States), careHPV (Qiagen, Gaithersburg, MD, United States), AmpFire (Atila BioSystems, United States), SeqHPV (BGI Shenzhen, Shenzhen, China), Cervista (Hologic, Marlborough, MA, United States), matrix-assisted laser desorption/ionization time-of-flight (MALDI-TOF, BGI Shenzhen, Shenzhen, China), and Cobas HPV test (Roche Molecular Systems, Inc., United States). Additionally, six other methods are introduced, including HPVDNA Chip™ (Biomedlab Co., Seoul, South Korea), PGMY PCR (Roche Molecular Systems, Inc., United States), Easy-Chip HPV Blot (King Car Yuanshan Research Institute, Taiwan, China), RealTime High Risk HPV assay (Abbott Molecular Inc., Abbott Park, IL), Anyplex II HPV kit assay (Seegene, Seoul, South Korea), and Linear Array HPV Genotyping test (Roche Diagnostics, United Kingdom). HC2, Cobas HPV test, and Cervista have received FDA/CE-IVD approval. HC2 test detects the presence of 13 HR-HPV types using full genome probes complementary to HPV DNA, specific antibodies, signal amplification, and chemiluminescent detection (Belinson et al., 2001). HPVDNA Chip uses HPV and β-globin primers to amplify the target HPV DNA through PCR under specific conditions, and the amplification products are labeled with Cy5-dUTP, which could contain 22 type-specific probes (15 for the high-risk group and seven the low-risk group) (Seo et al., 2006). PGMY PCR uses the PGMY09/11 L1 consensus primer system for PCR amplification and a reverse line blot detection strip that individually identifies 22 high-risk types (Bhatla et al., 2009). The careHPV assay, adapted from the HC2 assay, is a qualitative test for HR-HPV detection, targeting 14 HR-HPV types through hybridization of HR-HPV DNA with a cocktail of RNA probes and chemiluminescence signal amplification (Qiao et al., 2008). Easy-Chip HPV Blot contains 39 type-specific probes that are immobilized on a 14.4 mm × 9.6 mm nylon membrane, which is used for reverse-blot hybridization and detects HPV DNA in a single assay (Twu et al., 2011). Cervista is a signal-amplification method for the qualitative detection of 14 HR-HPV types (Belinson et al., 2012). MALDI-TOF is a mass spectrometry method that uses a multiplex primary PCR also for the same 14 HR-HPV types detected by Cervista (Belinson et al., 2012). Cobas HPV test is a real-time PCR assay that detects 14 HPV types, with HPV16 and HPV18 detected individually and the other 12 HPV types detected as a pooled group (Chen Q. et al., 2016b Terada et al., 2022). The AmpFire method is a nucleic acid amplification technique for qualitative detection of HR-HPV, using HR-HPV-specific primers and fluorescent probes to amplify the viral genomic DNA (including the E6/E7 region) under isothermal conditions. This method does not require DNA extraction or purification and can directly detect HPV from lysed clinical samples in one step (Zhang et al., 2020). The SeqHPV assay is a high-throughput HPV genotyping method based on multiplex PCR and next-generation sequencing, capable of detecting 14 HR-HPV types (Du et al., 2021). The Abbott m2000rt automatic biochemical analyzer was used for real-time fluorescence quantitative PCR detection. The detection boundary value of cycle threshold (CT) was 32.0, and the internal quality control target boundary value of CT was 35.0. Abbott HR-HPV assay could detect 14 HR HPV types (Abdullah et al., 2018; Aiko et al., 2017; Chen W. et al., 2014; Chen K. et al., 2016; Chou et al., 2016; Hariprasad et al., 2023; Li et al., 2022; Nutthachote et al., 2019; Page et al., 2021; Phoolcharoen et al., 2018a; Satake et al., 2020; Tan et al., 2021; Wong et al., 2020; Yoshida et al., 2011) simultaneously, and specifically identifies HPV16 and HPV18 (Qin et al., 2016). Anyplex^^TM^ II HPV 28 real-time PCR test simultaneously detects 19 HR-HPV and 9 low-risk HPV types, using dual priming oligonucleotides and a melting curve analysis method of tagging oligonucleotide cleavage and extension (Cho et al., 2019). Linear Array HPV Genotyping test (Roche Diagnostics, United Kingdom) combines consensus PCR and reverse-hybridization amplification products to detect 36 genital HPV genotypes. Because it has been clearly defined and validated in research and clinical applications, it is often considered the reference method for genital HPV genotyping (Yoshida et al., 2011).

### Quality assessment of included studies

**FIGURE 2:**
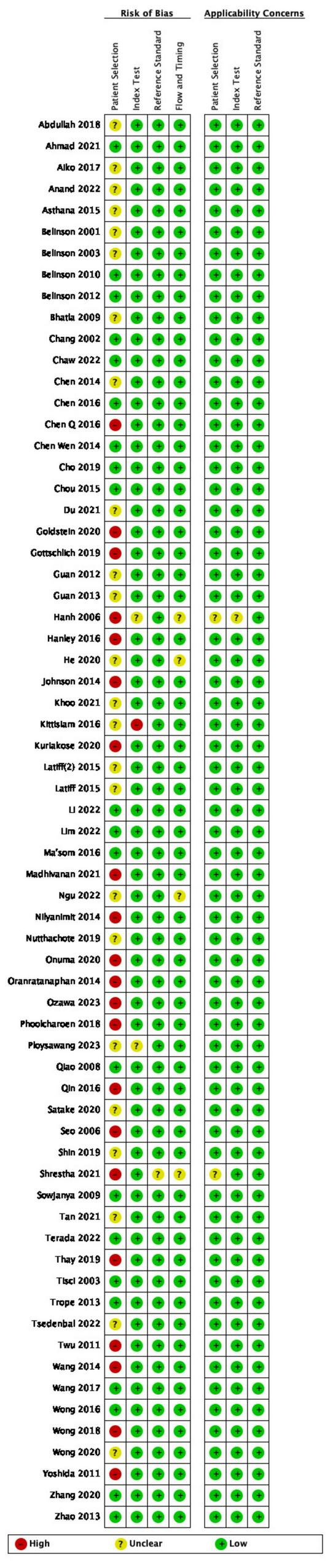
Quality assessment of included studies. Green, low risk of bias; red: high risk of bias; yellow, not reported/unclear risk of bias.

All the studies included in this systematic review were assessed for risk of bias (Figure 2). The Cohen’s kappa value between two independent reviewers was 0.839. Most of the studies included in the analysis were cross-sectional and did not employ random patient selection or allocation. As a result, the risk of bias in several domains was found to be high or unclear. Specifically, 21 studies were assessed as having a high risk of bias in the “Patient Selection” domain (Aiko et al., 2017; Chen Q. et al., 2016; Goldstein et al., 2020; Gottschlich et al., 2019; Hanh, 2006; Hanley et al., 2016; Johnson et al., 2014; Kuriakose et al., 2020; Madhivanan et al., 2021; Nilyanimit, 2014; Oranratanaphan et al., 2014; Ozawa et al., 2023; Phoolcharoen et al., 2018a; Qin et al., 2016; Seo et al., 2006; Shrestha et al., 2021; Thay et al., 2019; Twu et al., 2011; Wang et al., 2014; Wong et al., 2018; Yoshida et al., 2011). This high risk was attributed to the non-random selection of participants, which could introduce selection bias and limit the generalizability of the findings. One study was considered to have a high risk of bias in the “Index Testing” domain (Kittisiam et al., 2016), due to the use of a non-standardized or poorly validated diagnostic test, which could affect the accuracy of the results. In the “Reference Standard” domain, only one study was deemed to have an unclear risk of bias (Shrestha et al., 2021), due to a lack of detailed information regarding the reference standard used. In the “Flow and Timing” domain, four studies were assessed as having an unclear risk of bias (Hanh, 2006; He and He, 2020; Ngu et al., 2022; Shrestha et al., 2021), which was due to incomplete reporting of participant flow or unclear timing of tests, potentially leading to attrition or measurement bias. Notably, all studies were judged to have a low risk of diagnostic bias, as the diagnostic criteria were predefined prior to the availability of results, ensuring the objectivity of the assessment.

### Assessment of publication bias

**FIGURE 3:**
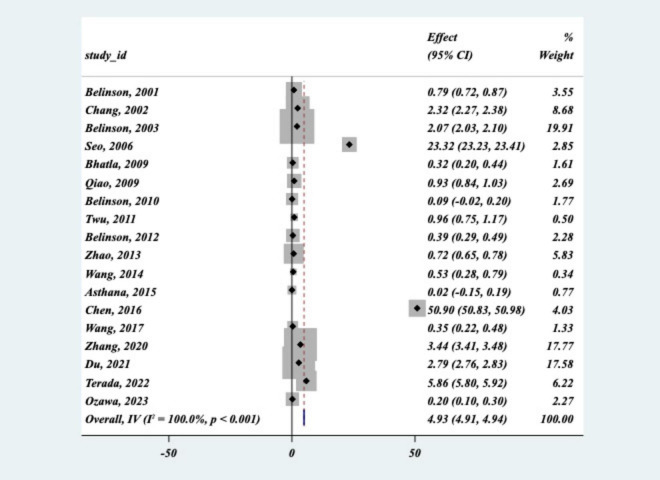
Funnel plots.

The Cochran’s Q statistic was highly significant (Q = 1.8 × 10^6^, *P* = 0.000), indicating substantial heterogeneity among the studies. Additionally, the I^2^ statistic was calculated to be 100%, suggesting that nearly all of the variability in effect sizes across studies could be attributed to differences between studies rather than random error. Begg’s test yielded a significant *p*-value (*P* < 0.05), further suggesting the presence of potential publication bias. This finding implies that smaller studies or studies with non-significant results may be underrepresented or unpublished, which could have influenced the overall effect size observed in the meta-analysis. The funnel plot (Figure 3) exhibited signs of asymmetry, suggesting the presence of publication bias. Specifically, there appears to be an over-representation of studies with larger effect sizes, while smaller studies with negative or null results may be underrepresented.

### Diagnostic accuracy of self-sampled HPV tests

Two studies found that the accuracy of HPV self-sampling is comparable to that of physician sampling (Aiko et al., 2017; Belinson et al., 2012). In the study of Belinson et al. (2012) when using the MALDI-TOF mass spectrometry system for HPV detection, the sensitivity of self-sampling for identifying CIN 3+ was equivalent to that of clinician sampling. However, when utilizing Cervista, the sensitivity for detecting CIN 3+ in self-collected specimens was only 70.9%, compared to 95.0% for clinician-collected samples (Belinson et al., 2012). In the study of Onuma et al. (2020) the sensitivity of HPV self-sampling and clinician sampling for the detection of CIN 2+ were both 100% (Aiko et al., 2017). In studies of Du et al. (2021), Zhang et al. (2020), the sensitivity for detecting CIN 2+ was higher in self-sampling than in clinician sampling. While in the other three studies of Belinson et al. (2001) the sensitivity for detecting CIN 3+ was 81% and 98% in self- and clinician-collected samples. In the remaining 12 studies, the sensitivity of HPV self-sampling was slightly lower than physician sampling, with values ranging from 59.4% to 87.5%, while the specificity of HPV self-sampling was identical to clinician sampling (Table 1).

**TABLE 1:** Sensitivity and specificity of two sampling methods in the diagnosis of CIN2+/CIN3+.

References	Number of patients	Methods of collection		Methods of test	CIN2+				CIN3+			
					**Sensitivity% (95%CI)**		**Specificity% (95%CI)**		**Sensitivity% (95%CI)**		**Specificity% (95%CI)**	
		**Self**	**Clinician**		**Self**	**Clinician**	**Self**	**Clinician**	**Self**	**Clinician**	**Self**	**Clinician**
Hariprasad et al., 2023	1,997	Dacron swab	Endocervical brush	HC2	82.56 (80.90–84.22)	95.35 (94.43–96.27)	85.92 (84.39–87.45)	85.24 (83.68–86.80)	81.39 (79.68–83.10)	97.67 (97.01–98.33)	84.39 (82.80–85.98)	83.52 (81.89–85.15)
Page et al., 2021	1,194	NR	NR	HC2	96.30 (95.23–97.37)	100 (0.849819–100)	91.80 (90.24–93.36)	91.06 (89.44–92.68)	NR	NR	NR	NR
Belinson et al., 2001	8,497	Conical shaped brush	Conical–shaped brush	HC2	87.50 (86.80–88.20)	96.80 (96.43–97.17)	77.20 (76.21–78.09)	79.70 (78.84–80.56)	NR	NR	NR	NR
Madhivanan et al., 2021	1,18	Dacron polyester swab	Dacron polyester swab	HPVDNAChip™	NR	NR	NR	NR	90.50 (85.21–95.79)	88.10 (82.26–93.94)	29.00 (20.81–37.19)	32.90 (24.42–41.38)
Tan et al., 2021	512	Digene HPV collection tube	Cervical brush sampler	HC2, PGMY PCR	82.50 (79.21–85.79) for PGMY PCR, 80.00 (84.64–90.36) for HC2	87.50 (84.64–90.36) for PGMY PCR, 90.00 (87.40–92.60) for HC2	93.64 (91.53–95.75) for PGMY PCR, 88.14 (85.34–90.94) for HC2	93.22 (91.04–95.40) for PGMY PCR, 91.74 (89.36–94.12) for HC2	NR	NR	NR	NR
Belinson et al., 2003	2,388	Vaginal–brush specimen	Cervical brush	careHPV	81.40 (79.84–82.96)	90.50 (89.32–91.68)	82.40 (80.87–83.93)	84.20 (82.74–85.56)	82.60 (81.08–84.12)	87.00 (85.65–88.35)	81.10 (79.53–82.67)	82.70 (81.18–84.22)
Tisci et al., 2003	2,653	Conical-shaped brush	Conical-shaped brush	HC2	80.90 (79.40–82.40)	88.60 (87.39–89.81)	97.90 (97.35–98.45)	90.20 (89.07–91.33)	NR	NR	NR	NR
Qiao et al., 2008	252	Cytobrush	Endocervical brush	EasyChip HPV Blot	NR	NR	NR	NR	75.00 (69.65–80.35)	87.50 (83.42–91.58)	75.80 (70.51–81.09)	73.70 (68.26–79.14)
Belinson et al., 2010	8,556	POI/NIH self-sampler, conical-shaped brush	Rovers Cervex brush	Cervista, MALDI-TOF	NR	NR	NR	NR	70.92 (69.96–71.88) for cervista, 94.33 (93.84–94.82) for MALDI-TOF	95.04 (94.58–95.50) for cervista, 94.33 (93.84–94.82) for MALDI-TOF	86.13 (85.40–86.86) for cervista, 87.58 (86.88–88.28) for MALDI-TOF	90.29 (89.66–90.92) for cervista, 89.44 (88.79–90.09) for MALDI-TOF
Guan et al., 2012	7,543	NR	Polyester swab	careHPV, HC2	82.6 (75.4–88.4) for careHPV, 91.7 (85.9–95.6) for HC2	95.8 (91.2–98.5) for careHPV and HC2	86.9 (86.1–87.7) for careHPV, 83.6 (82.7–84.4) for HC2	87.3 (86.5–88.1) for careHPV, 87.1 (86.3–87.9) for HC2	83.8 (75.1–90.5) for careHPV, 90.9 (83.4–95.8) for HC2	97.0 (91.4–95.8) for careHPV and HC2	86.5 (85.7–87.2) for careHPV, 83.1 (82.2–83.9) for HC2	86.8 (86.0–87.6) for careHPV, 86.6 (85.8–87.4) for HC2
Chen S. et al., 2014	396	Conical Cervical Sampler	NR	careHPV	66.70 (62.06–71.34)	83.30 (79.63–86.97)	79.00 (74.99–83.01)	77.90 (73.81–81.99)	NR	NR	NR	NR
Bhatla et al., 2009	4,658	care HPV sampler	care HPV sampler	careHPV	40.60 (39.19–42.01)	53.10 (51.67–54.53)	97.30 (96.83–97.77)	97.75 (97.32–98.18)	53.80 (52.37–55.23)	84.60 (83.56–85.64)	97.55 (97.11–97.99)	97.30 (96.83–97.77)
Ngu et al., 2022	197	Cone-shaped brush	Cone-shaped brush	Cobas 4800 HPV assay	92.86 (89.26–96.46)	95.24 (92.27–98.21)	20.35 (14.73–25.97)	16.81 (11.59–22.03)	96.00 (93.26–98.74)	98.00 (96.04–99.96)	18.37 (12.96–23.78)	14.97 (9.99–19.95)
Chen K. et al., 2016	2,337	Vaginal-brush specimen	Cervical brush	careHPV	72.10 (70.28–73.92)	83.80 (82.31–85.29)	88.20 (86.89–89.51)	88.10 (86.79–89.41)	NR	NR	NR	NR
Terada et al., 2022	100	Evalyn Brush	Rovers Cervex brush	Cobas 4800 HPV assay	100	100	58.10 (48.43–67.77)	57.00 (47.30–66.70)	NR	NR	NR	NR
Goldstein et al., 2020	6,042	“JustForMe” brush	Broom sampler	AmpFire HPV assay	96.81 (96.37–97.25)	95.74 (95.23–96.25)	89.81 (89.05–90.57)	90.77 (90.04–91.50)	100	100	89.01 (88.22–89.80)	89.98 (89.22–90.74)
He and He, 2020	10,339	“JustForMe” brush	Broom sampler	Cobas 4800 HPV assay, SeqHPV assay	95.07 (94.65–95.49) for Cobas 4800, 96.48 (96.12–96.84) for Seq HPV	95.07 (94.65–95.49) for Cobas 4800, 93.66 (93.19–94.83) for Seq HPV	87.35 (86.71–87.99) for Cobas 4800, 89.53 (88.84–90.02) for Seq HPV	90.38 (89.81–90.95) for Cobas 4800, 90.25 (89.68–90.82) for Seq HPV	96.30 (95.94–96.66) for Cobas 4800, 100 (91.73–100) for Seq HPV	100 for Cobas 4800, 100 (91.73–100) for Seq HPV	86.65 (85.59–87.31) for Cobas 4800, 88.82 (88.22–89.44) for Seq HPV	89.69 (89.10–90.28) for Cobas 4800, 89.57 (8.98–90.16) for Seq HPV
Aiko et al., 2017	300	Evalyn Brush	NR	Cobas 8800 system	84.80 (80.74–88.86)	89.10 (85.57–92.63)	48.77 (43.11–54.43)	38.89 (33.37–44.41)	89.61 (86.16–93.06)	90.91 (87.66–94.16)	44.39 (38.77–50.01)	31.84 (26.57–37.11)
Satake et al., 2020	165	Home Smear Set Plus	Cervex brush	Cobas 4800 HPV assay	81.40 (75.46–87.34)	89.80 (85.18–94.42)	NR	NR	NR	NR	NR	NR

HPV, human papillomavirus; HC2, hybrid capture II; PCR, polymerase chain reaction; CIN, cervical intraepithelial neoplasia; NR, data not reported; CI, confidence interval.

In the detection of HPV, 17 studies reported the detection rates were higher in clinician sampling (Anand et al., 2022; Asthana and Labani, 2015; Belinson et al., 2001; Chang et al., 2002; Chen K. et al., 2016; Kuriakose et al., 2020; Latiff et al., 2015a; Madhivanan et al., 2021; Ma’som et al., 2016; Satake et al., 2020; Seo et al., 2006; Singh et al., 2023; Terada et al., 2022; Thay et al., 2019; Twu et al., 2011; Wang et al., 2014; Wang et al., 2017), while in 15 studies this rate was higher in self-collected samples (Belinson et al., 2003; Cho et al., 2019; Du et al., 2021; Latiff et al., 2015a; Nutthachote et al., 2019; Qin et al., 2016; Satake et al., 2020; Seo et al., 2006; Terada et al., 2022; Thay et al., 2019; Wong et al., 2016; Wong et al., 2018; Yoshida et al., 2011; Zhang et al., 2020; Zhao et al., 2013). Two studies evaluated that detection rates of both sampling methods were the same (Anand et al., 2022; Nilyanimit, 2014). In 13 studies, the difference in detection rates was not more than 1% (Aiko et al., 2017; Asthana and Labani, 2015; Belinson et al., 2001; Bhatla et al., 2009; Chang et al., 2002; Chen W. et al., 2014; Hanh, 2006; Kuriakose et al., 2020; Lim et al., 2022; Satake et al., 2020; Wang et al., 2017; Zhang et al., 2020; Zhao et al., 2013; Table 2). In the studies by Wong and Yoshida et al., multiple types of HPV infections were found to occur more frequently with self-sampling compared to clinician sampling (Wong et al., 2016; Yoshida et al., 2011).

**TABLE 2:** HPV detection rate of two sampling methods.

References	Sample size	Methods of collection		Methods of test	HPV detection rate(95% CI)	
		**Self**	**Clinician**		**Self**	**Clinician**
Hariprasad et al., 2023	1997	Dacron swab	Endocervical brush	HC2	17% (15.35–18.65%)	18% (16.31–19.69%)
Belinson et al., 2001	8,497	Conical shaped brush	Conical-shaped brush	HC2	25.60% (24.67–26.53%)	23.71% (22.81–24.61%)
Wang et al., 2014	392	Dacron swab	Cytobrush	PCR	11.70% (8.52–14.88%)	7.70% (5.06–10.34%)
Qin et al., 2016	68	Dacron swab	Dacron swab	PGMY PCR	39.70% (28.07–51.33%)	36.80% (25.34–48.26%)
Lim et al., 2022	250	NR	NR	careHPV	22.40% (17.23–27.57%)	18.00% (13.24–22.76%)
Kittisiam et al., 2016	400	Brush type collecting system	Broom type cervicalbrush	HC2	10.00% (7.06–12.94%)	7.50% (4.92–10.08%)
Hanley et al., 2016	300	Home Smear Set	Rovers Cervex brush	Cobas 4800 HPV assay	14.70% (10.69–18.71%)	13.70% (9.81–17.59%)
Ahmad et al., 2021	432	Vaginal self–swab sample	Digene cervical sampler	HC2, PCR	14.1% (10.82–17.38%) with HC2, 16.4% (12.91–19.89%) with PCR	20.1% (16.32–23.88%) with HC2, 20.6% (16.79–24.41%) with PCR
Qiao et al., 2008	252	Cytobrush	Endocervical brush	EasyChip HPV Blot	27.40% (21.89–32.91%)	30.20% (24.53–35.87%)
Ozawa et al., 2023	486	Kato self-samplingdevise	Cytobrush	PCR	17.30% (13.94–20.66%)	23.90% (20.11–27.69%)
Onuma et al., 2020	258	Cervisafe Self-sampling device	Endocervical brush	PGMY PCR	5.81% (2.96–8.66%)	3.87% (1.52–6.22%)
Shrestha et al., 2021	300	NR	NR	Cobas 6800 HPV assay	20.00% (15.47–24.53%)	21.00% (16.39–25.61%)
Asthana and Labani, 2015	120	Broom-type collection device	Brush-like collection device	HC2	10.10% (4.71–15.49%)	12.60% (6.66–18.54%)
Tan et al., 2021	512	Digene HPV collection tube	Cervical brush sampler	HC2, PGMY PCR	12.30% (9.46–15.14%) with PGMY PCR, 14.60% (11.54–17.66%) with HC2	13.10% (10.18–16.02%) with PGMY PCR, 17.20% (13.93–24.07%) with HC2
Phoolcharoen et al., 2018b	136	Evalyn brush	Cytopic device	HC2	40.40% (32.15–48.65%)	61.00% (52.80–69.20%)
Satake et al., 2020	165	Home Smear Set Plus	Cervex brush	Cobas 4800 HPV assay	59.39% (51.90–66.88%)	62.42% (55.03–69.81%)
Ploysawang et al., 2023	50	Rovers Viba-brush	Rovers Cervex brush	Linear array HPV Genotyping test	82.00% (71.35–92.65%)	74.00% (61.84–86.16%)
Terada et al., 2022	100	Evalyn brush	Rovers Cervex brush	Cobas 4800 HPV assay	50.00% (40.20–59.80%)	51.00% (41.2–60.8%)
Sowjanya et al., 2009	114	Sterile swab with ascrew cap	NR	PGMY PCR	77.20% (69.5–84.9%)	78.10% (70.51–89.69%)
Trope et al., 2013	101	Flexible minitip flocked swab	Flexible minitip flocked swab	PGMY PCR	40.60% (31.02–50.18%)	40.60% (31.02–50.18%)
Zhao et al., 2013	7,541	NR	NR	careHPV	14.69% (13.89–15.49%) for careHPV, 15.05% (14.24–15.86%) for HC2	14.97% (14.16–15.78%) for careHPV, 18.53% (17.65–19.41%) for HC2
Page et al., 2021	1,194	NR	NR	HC2	12.10% (10.25–13.95%)	13.00% (11.09–14.91%)
Tsedenbal et al., 2022	1238	NR	NR	NR	3.86% (2.79–4.93%)	4.05% (2.95–5.15%)
Madhivanan et al., 2021	118	Dacron polyester swab	Dacron polyester swab	HPVDNAChip™	90.50% (85.21–95.79%)	88.10% (82.26–93.94%)
Guan et al., 2012	7543	NR	Polyester swab	careHPV	14.5% (13.71–15.29%) for careHPV, 17.9% (17.03–18.77%) for HC2	14.4% (13.61–15.19%) for careHPV, 14.5% (13.71–15.29%) for HC2
Chen S. et al., 2014	396	Conical cervical sampler	NR	careHPV	22.49% (18.38–26.60%)	24.01% (19.80–28.22%)
Bhatla et al., 2009	4,658	care HPV sampler	care HPV sampler	careHPV	2.40% (1.96–2.84%)	2.90% (2.42–3.38%)
Ngu et al., 2022	197	Cone-shaped brush	Cone–shaped brush	Cobas 4800 HPV assay	85.28% (80.33–90.23%)	88.32% (83.83–92.81%)
Wong et al., 2016	291	Conical brush	Broom brush	RealTime high risk HPV assay	42.61% (36.93–48.29%)	36.86% (31.32–42.40%)
Chen K. et al., 2016	2337	Vaginal-brush specimen	Cervical brush	careHPV	13.60% (12.21–14.99%)	14.00% (12.59–15.41%)
Anand et al., 2022	101	Flocked swab	Cervical brush	Anyplex II HPV 28, Cobas 4800, RealTime HR-S HPV	86.10% (79.35–92.85%) for RealTime HR-S, 88.10% (81.79–94.41%) for Anyplex II, 88.10% (81.79–94.41%) for Cobas 4800	83.20% (75.41–90.49%) for RealTime HR-S, 80.20% (72.43–87.97%) for Anyplex II, 78.20% (70.15–86.25%) for Cobas 4800
Goldstein et al., 2020	6,042	“JustForMe” brush	Broom sampler	AmpFire HPV assay	11.50% (10.70–12.30%)	10.60% (9.82–11.38%)
He and He, 2020	10,399	“JustForMe” brush	Broom sampler	CobaS 4800 HPV assay, SeqHPV assay	13.80% (13.14–14.46%) for Cobas 4800, 11.60% (10.98–12.22%) for Seq HPV	10.80% (10.20–11.40%) for Cobas 4800, 10.90% (10.30–11.50%) for Seq HPV
Kuriakose et al., 2020	1,000	NR	NR	HC2	2.70% (1.70–3.70%)	2.70% (1.70–3.70%)
Aiko et al., 2017	300	Evalyn brush	NR	Cobas 8800 system	74.00% (69.04–78.96%)	66.67% (61.34–72.00%)

HPV, human papillomavirus; HC2, hybrid capture II; PCR, polymerase chain reaction; NR, data not reported; CI, confidence interval.

### Concordance between self-sampling and clinician sampling or cytology for HR-HPV

A total of 29 studies reported an agreement between HPV self-sampling and clinician sampling. A total of 24 reported a high or nearly perfect agreement between self-sampling and clinician sampling for the detection of HPV DNA. Specifically, 18 studies demonstrated an agreement exceeding 90% (Anand et al., 2022; Bhatla et al., 2009; Chen K. et al., 2016; Chen Q. et al., 2016; Du et al., 2021; Johnson et al., 2014; Kuriakose et al., 2020; Latiff et al., 2015a; Madhivanan et al., 2021; Ngu et al., 2022; Nilyanimit, 2014; Nutthachote et al., 2019; Satake et al., 2020; Seo et al., 2006; Sowjanya et al., 2009; Tsedenbal et al., 2022; Wang et al., 2014; Wong et al., 2016). Three studies assessed the agreement in both collecting methods samples using two assays for the detection of HPV (Chen W. et al., 2014; Du et al., 2021; Sowjanya et al., 2009), and one study evaluated the concordance of both sampling methods in three HPV testing assays (Cho et al., 2019). Some new HPV assays such as SeqHPV and careHPV showed higher agreement in self- and clinician-collected samples. In studies of Chen W. et al. (2014), Du et al. (2021) when the same sample was tested using different detection methods, the consistency of clinician-sampled samples was higher than that of self-sampled samples.

However, three studies have reported poor agreement between self- and clinician sampling results for the detection of HPV. Twu et al. (2011) reported low agreement between vaginal and cervical specimens using the EasyChip HPV Blot (k = 0.37) (Table 3).

**TABLE 3:** Concordance between HPV results from self-collected and clinician-collected samples.

References	Number of patients	Method of collection		Agreement rate	Cohen’s kappa (95% CI)
		**Self**	**Clinician**		
Anand et al., 2022	118	Dacron polyester swab	Dacron polyester swab	93.22% (88.68–97.76%) for HPVDNAChip™	0.82 (0.69–0.94) for HPVDNAChip™
Tan et al., 2021	512	Pre-labeled Digene HPV collection tube	Endocervical brush	93.75% (91.65–95.85%)	0.76 (0.64–0.82)
Ahmad et al., 2021	432	Vaginal self-swab sample	Digene cervical sampler	92.59% (90.12–95.06%)	0.76 (0.72–0.89)
Ploysawang et al., 2023	50	Rovers Viba-brush vaginal sampler	Rovers Cervex-brush	84.00% (72.93–95.07%)	0.54 (0.24–0.83)
Qiao et al., 2008	252	Cytobrush	Endocervical cytobrush	74.20% (68.80–79.60%)	0.37 (0.25–0.50)
Shin et al., 2019	261	APTIMA Cervical Specimen Collection and Transport (CSCT) kit	APTIMA Cervical Specimen Collection and Transport (CSCT) kit	95.02% (92.38–97.66%)	0.62 (0.43–0.81)
Trope et al., 2013	101	Flexible minitip flocked swab	Flexible minitip flocked swab	92.08% (86.81–97.35%)	0.83 (0.72–0.95)
Zhao et al., 2013	7,543	NR	NR	90.31% (89.64–90.98%) for HC2, 91.08% for careHPV	0.65 (0.63–0.67) for HC2, 0.64 (0.61–0.67) for careHPV
Ozawa et al., 2023	226	Kato self-sampling device	Pap smear cytobrush	86.28% (81.79–90.77%)	0.64 (0.53–0.75)
Onuma et al., 2020	258	Cervisafe^®^ device	Endocervical brush with detachable tip	98.06% (96.38–99.74%)	0.71 (0.44–0.98)
Wang et al., 2014	392	Dacron swab	Pap smear cytobrush	93.88% (95.02–99.76%)	0.65 (0.51–0.78)
Chou et al., 2016	202	Evalyn Brush	Digene Female Swab Specimen Collection Kit	97.52% (95.38–99.66%)	0.95 (0.91–0.99)
Ngu et al., 2022	197	Cone-shaped brush (Qiagen, Venlo, Netherlands)	Cone-shaped brush (Qiagen, Venlo, Netherlands)	94.92% (91.85–97.99%)	0.78 (0.65–0.91)
Wong et al., 2016	291	Conical brush (Qiagen, Gaithersburg, United States)	Broom brush	86.94% (83.07–90.81%)	0.73 (0.65–0.81)
Yoshida et al., 2011	136	Evalyn Brush	Digene HC2 DNA Collection device	77.94% (67.04–81.50%)	0.59 (0.46–0.72)
Qin et al., 2016	68	Dacron swab	Dacron swab	85.29% (76.87–93.71%)	0.69 (0.51–0.87)
Nilyanimit, 2014	247	Evalyn brush	Rovers Cervex-brush	74.49% (69.05–79.93%)	0.46 (0.36–0.56)
Kittisiam et al., 2016	400	Brush type collecting system (QIAGEN Gaithersburg, Inc.)	Broom type cervicalbrush (Surepath^®^)	95.50% (93.47–97.53%)	0.73 (0.60–0.86)
Anand et al., 2022	101	Flocked Swab (Noble Biosciences, Inc., Gyeonggi-Do, South Korea)	Cervical Brush (Noble Biosciences, Inc., Gyeonggi-Do, South Korea)	89.1% (83.02–95.18%) for RealTime HR-S, 86.1% (79.35–92.85%) for Anyplex II, 73.3% (64.67–81.93%) for Cobas 4800	0.58 (0.36–0.80) for RealTime HR-S, 0.49 (0.26–0.71) for Anyplex II, 0.51 (0.30–0.73) for Cobas 4800
Terada et al., 2022	100	Evalyn brush	Rovers Cervex-brush	88.00% (81.63–94.37%)	0.76 (0.69–0.82)
Hanley et al., 2016	300	Rovers Cervex-brush	Rovers Cervex-brush	96.33% (94.20–98.46%)	0.85 (0.76–0.94)
Sowjanya et al., 2009	114	Sterile swab with screw cap.	NR	93.85% (89.44–98.26%)	0.82 (0.64–1.00)
Asthana and Labani, 2015	120	Digene HC2 NA Collection device	Digene HC2 NA Collection device	94.12% (89.91–98.33%)	0.73 (0.34–1.00)
He and He, 2020	10,339	“Just For Me” brush (CE-marked; Preventive Oncology International, Inc, Cleveland Heights, OH).	Broom sampler (Rovers Medical Devices, Oss, Netherlands)	95.13% (94.72-95.54%) for SeqHPV, 95.13% (94.72–95.54%) for Cobas 4800	0.91 (0.89–0.92) for SeqHPV, 0.77 (0.76–0.79) for Cobas 4800
Zhang et al., 2020	121	Dacron swab	Cervex-brush	90.2% (85.1–93.8%)	0.59 (0.42–0.75)
Shrestha et al., 2021	171	Flocked swab	NR	92.33% (88.34–96.32%)	0.77 (0.67–0.86)
Kuriakose et al., 2020	1,000	NR	NR	95.11% (93.77–96.45%)	0.57 (0.40–0.73)
Aiko et al., 2017	300	NR	NR	58.67% (53.10–64.24%)	0.77 (0.69–0.85)
Satake et al., 2020	165	HPV self-sampling kit using sponge device (HSD-ST)	Cervex Brush^®^ (Becton, Dickinson, and Company)	88.48% (82.6–92.9%)	0.76 (0.66–0.86)

HPV, human papillomavirus; Pap, Papanicolaou cytology; HC2, hybrid capture II; PCR, polymerase chain reaction; NR, data not reported; CI, confidence interval.

### Acceptability of self-collection for HPV testing

A total of 29 studies have assessed women’s overall acceptance of HPV self-sampling. The lowest reported acceptance was 40.3% (95% CI: 38.49%–42.11%) (Trope et al., 2013), while the highest reached 100% (Anand et al., 2022; Ploysawang et al., 2023). In 27 of the 29 studies, acceptance exceeded 60% (Abdullah et al., 2018; Ahmad et al., 2021; Aiko et al., 2017; Anand et al., 2022; Chen S. et al., 2014; Cho et al., 2019; Chou et al., 2016; Goldstein et al., 2020; Gottschlich et al., 2019; Guan et al., 2012; Hanley et al., 2016; Khoo et al., 2021; Kittisiam et al., 2016; Li et al., 2022; Ma’som et al., 2016; Ngu et al., 2022; Oranratanaphan et al., 2014; Phoolcharoen et al., 2018b; Ploysawang et al., 2023; Shrestha et al., 2021; Singh et al., 2023; Sowjanya et al., 2009; Thay et al., 2019; Tisci et al., 2003; Trope et al., 2013; Wong et al., 2016; Wong et al., 2020). A total of 13 studies indicated that women preferred self-sampling over clinician sampling (Goldstein et al., 2020; Gottschlich et al., 2019; Hanh, 2006; Khoo et al., 2021; Li et al., 2022; Lim et al., 2022; Madhivanan et al., 2021; Ploysawang et al., 2023; Shin et al., 2019; Shrestha et al., 2021; Trope et al., 2013; Wong et al., 2016; Wong et al., 2018), however, three studies found a preference for clinician sampling instead (Aiko et al., 2017; Ngu et al., 2022; Tsedenbal et al., 2022; Table 4).

**TABLE 4:** Acceptability of self-collection for HPV testing.

References	Sample size	Acceptability (95% CI)	Preference for self-sampling (95% CI)	Preference for physician-sampling (95% CI)
Chang et al., 2002	1,560	100%	NR	NR
Tsedenbal et al., 2022	1,216	NR	42.08% (39.31–44.85%)	41.04% (38.27–43.80%)
Shin et al., 2019	432	99.96% (99.77–100.14%)	NR	NR
Belinson et al., 2012	174	86.60% (81.54–91.66%)	NR	74.00% (67.48–80.52%)
Li et al., 2022	431	90.50% (87.73–93.27%)	71.30% (67.03–75.57%)	9.80% (6.99–12.61%)
Guan et al., 2013	297	66.00% (60.61–71.39%)	NR	NR
Chen Q. et al., 2016	100	79.00% (71.02–86.98%)	NR	NR
Chen W. et al., 2014	282	90.80% (87.43–94.17%)	65.20% (59.64–70.76%)	NR
Latiff et al., 2015a	839	91.80% (89.94–93.66%)	68.20% (65.05–71.35%)	NR
Phoolcharoen et al., 2018b	200	90.00% (85.84–94.16%)	NR	NR
Oranratanaphan et al., 2014	2,810	40.30% (38.49–42.11%)	NR	NR
Wang et al., 2014	392	77.00% (83.34–90.06%)	56.90% (52.00–61.80%)	37.80% (33.00–42.60%)
Yoshida et al., 2011	136	86.70% (80.99–92.41%)	45.70% (37.33–54.07%)	54.30% (45.93–62.67%)
Qin et al., 2016	64	NR	65.60% (53.96–77.24%)	34.40% (22.76–46.04%)
Latiff et al., 2015b	164	93.20% (89.35–97.05%)	NR	NR
Nilyanimit, 2014	247	80.80% (75.89–85.71%)	NR	NR
Phoolcharoen et al., 2018a	264	100%	69.86% (64.32–75.40%)	2.74% (0.77–4.71%)
Seo et al., 2006	728	93.41% (91.61–95.21%)	51.99% (48.36–55.62%)	24.07% (20.96–27.18%)
Ma’som et al., 2016	725	99.90% (99.67–100.13%)	83.00% (80.27–85.73%)	5.00% (3.41–6.59%)
Wang et al., 2017	177	72.88% (66.33–79.43%)	69.35% (61.24–77.46%)	NR
Wong et al., 2018	600	96.83% (95.43–98.23%)	64.83% (61.01–68.65%)	35.17% (31.35–38.99%)
Wong et al., 2020	1,810	42.32% (40.04–44.60%)	NR	NR
Abdullah et al., 2018	55	NR	40.00% (9.64–70.36%)	NR
Khoo et al., 2021	220	84.54% (79.76–89.32%)	NR	NR
Cho et al., 2019	30	NR	56.67% (38.94–74.40%)	43.33% (25.60–61.06%)
Asthana and Labani, 2015	120	100.00%	59.30% (50.51–68.09%)	28.00% (19.97–36.03%)
Johnson et al., 2014	175	88.89% (84.23–93.55%)	36.00% (28.89–43.11%)	64.00% (56.89–71.11%)
Thay et al., 2019	97	94.80% (90.26–99.34%)	NR	54.60% (44.69–64.51%)
Zhang et al., 2020	316	89.20% (85.78–2.62%)	32.80% (27.62–37.98%)	39.52% (34.13–44.91%)
Shrestha et al., 2021	300	90.00% (86.61–93.39%)	84.00% (79.85–88.15%)	13.00% (9.19–16.81%)
Kuriakose et al., 2020	1,000	97.00% (95.94–98.06%)	NR	NR
Du et al., 2021	8,136	95.97% (95.54–96.40%)	62.37% (61.32–63.42%)	37.63% (36.58–38.68%)
Nutthachote et al., 2019	265	93.58% (90.63–95.53%)	66.42% (60.73–72.11%)	33.58% (27.89–39.27%)

NR, data not reported; CI, confidence interval.

When asked about their preferred location for self-sampling, four studies found that participants preferred to perform the test at the clinic rather than at home (Belinson et al., 2001; Chen K. et al., 2016; Kittisiam et al., 2016; Zhang et al., 2020). In contrast, three studies reported a preference for sampling at home (Onuma et al., 2020; Seo et al., 2006; Tan et al., 2021).

## Discussion

Cervical cancer remains the leading cause of cancer death in Asia, especially South-Eastern Asia. China and India account for more than 50% of new cases of cervical cancer globally (Singh et al., 2023). Given that most cervical cancers are caused by persistent infection with high-risk HPV types, increasing participation in HPV-based cervical cancer screening is essential to reduce cervical cancer incidence. As a major screening method for cervical cancer, HPV self-sampling was recommended by WHO and other organizations (Simelela, 2021). However, the participation rate of cervical cancer screening in Asian women is still far below 70% and varies widely among different regions (Ong et al., 2023).

This systematic review analyzed the accuracy, agreement, and acceptability of HPV self-sampling in Asia. Though remains slightly lower than that of clinician sampling, the sensitivity and specificity of HPV self-sampling to detect CIN2+ is high, ranging from 60% to 100%. However, in some studies, the sensitivity of self-sampling was identical to or higher than that of clinician sampling for DNA testing, especially when researchers used new collection devices, such as the “JustForMe” brush and Dacron polyester swab. There was excellent agreement between the two sampling methods in the majority of studies, which was the same to the results of two systematic reviews in Africa and low-income countries (Kamath Mulki and Withers, 2021; Nodjikouambaye et al., 2020). These observations suggest that the quantity and quality of cervicovaginal exfoliated cells obtained by patients themselves are comparable to those obtained by physicians. In a study conducted in India, the sensitivity of self-sampling was found to be only 40.6%, while the specificity was 97.3%. Besides, the concordance between the two sampling methods, were notably low in some studies. This phenomenon may be attributed to various factors, including whether women correctly understood the process of self-sampling, differences in sampling techniques, sample quality and collection methods, HPV testing methods and diagnostic thresholds. While methods such as SeqHPV, careHPV, and RealTime HR-S have demonstrated high detection rates in some studies, the EasyChip HPV Blot has shown lower detection rates in self-collected samples. We recognize that non-standardized methods may not provide the same level of reliability and performance as WHO-approved tests. Specifically, these methods can present challenges related to sensitivity, specificity, reproducibility, and ease of use. In the absence of extensive validation and standardization, such methods may exhibit significant variability in results, which can undermine diagnostic accuracy. Therefore, standardized testing methods are essential to ensure that cervical cancer screening remains both accurate and reliable across different healthcare settings. It is expected to improve the accuracy of self-sampling by enhancing sampling instruments and testing methods, as well as increasing women’s understanding of the self-sampling process.

The study participants reported broad acceptance of self-sampling, and preferred self-sampling over clinician sampling, particularly among women with higher education and greater knowledge of HPV. Asia, comprising 44 countries, is characterized by its diverse cultures, religious beliefs, economic conditions, and medical practices. Factors such as a lack of understanding of HPV, cultural barriers, and limited economic and medical resources may hinder women’s participation in screening programs. Many women expressed a lack of confidence in self-sampling at home due to concerns about the reliability of self-collected samples without a doctor’s guidance and misunderstandings regarding the results. They also emphasized the need for timely follow-up and explanations of HPV test results. By promoting awareness of HPV and cervical cancer and educating patients about the importance of cervical cancer screening, we can improve the acceptance of HPV self-sampling among patients.

Human papillomavirus self-sampling can effectively increase cervical cancer screening participation rates among women, especially among women who have never been screened or are under-screened due to feeling embarrassed. The main reasons for low acceptability were that the participants were unaware of the relationship between HPV and cervical cancer, worried that self-sampling was not reliable, did not have access to consult a doctor, or did not understand the procedure of self-sampling. Women are more willing to perform self-HPV sampling at clinics or community health centers, highlighting a significant need for professional health workers to explain the self-sampling process, interpret test results, and provide follow-up support. Nevertheless, only ten countries in Asia have reported results from studies on HPV self-sampling, indicating that the coverage of this practice remains low. In addition, HPV vaccination rates are closely associated with cervical cancer screening uptake. HPV-unvaccinated women are generally less engaged in screening compared to those who have been vaccinated (Taniguchi et al., 2019). Moreover, inadequate healthcare infrastructure remains a significant barrier to the effective implementation of cervical cancer screening, particularly in many parts of Asia (Rajkhowa et al., 2024). Increased financial support, improved HPV vaccination rates, and healthcare professionals and infrastructures are essential to advance the cervical cancer elimination plan proposed by the WHO.

Currently, the use of urine and menstrual blood self-sampling for cervical cancer screening has been explored (Martinelli Li et al., 2022; Wong et al., 2010), but the available data are still insufficient. Efforts to enhance participation in cervical cancer screening and ensure timely treatment of precancerous lesions will contribute to reducing and ultimately eliminating cervical cancer.

### Limitations

This review is not without limitations. The characteristics of the participants enrolled in the primary studies differed, as did the sample sizes. In addition, the methods for HPV testing and sampling devices were not described in several studies. The diagnostic accuracy of the HPV tests was not uniform across the studies, and the intervals between self-sampling and clinician sampling were also inconsistent. Another limitation of this review is that only studies conducted in East Asia, Southeast Asia, and South Asia were included. Finally, gray literature and conference abstracts were not included in this review, and the exclusion of non-English articles may have limited the comprehensiveness of the analysis. These exclusions could introduce potential bias, as studies published in languages other than English or in gray literature might have different characteristics or findings compared to those published in peer-reviewed journals. Consequently, the findings of this review may not fully represent the entire body of literature, and future research should consider including non-English studies and gray literature to provide a more comprehensive understanding of the topic.

## Conclusion

Self-sampling for HPV detection can significantly improve cervical cancer screening coverage, especially in regions with limited medical resources or among individuals unwilling to undergo physician-collected sampling. However, its effectiveness varies across regions due to cultural, infrastructural, and healthcare factors. In rural areas of China and India, studies show that self-sampling accuracy is lower than physician-collected samples, likely due to differences in viral load capture and diagnostic thresholds. The diagnostic criteria and HPV testing methods for self-collected samples still need to be adjusted. Additionally, acceptance of self-sampling is low in China and Thailand, particularly among older women in these regions, due to concerns about procedure discomfort, infection, and reliability. To address these issues, targeted education and awareness campaigns are essential. Given these regional differences, self-sampling should be integrated into screening programs based on local contexts: in high-resource settings, physician-collected samples may remain preferred, while in low-resource areas, self-sampling can play a crucial role in expanding coverage. Policymakers should consider regional variations in healthcare infrastructure, cultural factors, and screening barriers to effectively reduce cervical cancer burden across diverse populations.

## Data Availability

The original contributions presented in this study are included in the article/Supplementary material, further inquiries can be directed to the corresponding author.
