# Supplementary material for: Human papillomavirus self-sampling in Asia: a systematic review
Source: Front Microbiol. 2025 Mar 14;16:1540609. doi: 10.3389/fmicb.2025.1540609 (PMC11949917; doi:10.3389/fmicb.2025.1540609)
Supplement: Supplementary file 1 [file Data_Sheet_1.docx]

**Table S1: PRISMA checklist**

| **Section and Topic** | **Item #** | **Checklist item** | **Location where item is reported** |
| --- | --- | --- | --- |
| **TITLE** | | |  |
| Title | 1 | Identify the report as a systematic review. | 1, 2 |
| **ABSTRACT** | | |  |
| Abstract | 2 | See the PRISMA 2020 for Abstracts checklist. | 2 |
| **INTRODUCTION** | | |  |
| Rationale | 3 | Describe the rationale for the review in the context of existing knowledge. | 3 |
| Objectives | 4 | Provide an explicit statement of the objective(s) or question(s) the review addresses. | 3 |
| **METHODS** | | |  |
| Eligibility criteria | 5 | Specify the inclusion and exclusion criteria for the review and how studies were grouped for the syntheses. | 3 |
| Information sources | 6 | Specify all databases, registers, websites, organisations, reference lists and other sources searched or consulted to identify studies. Specify the date when each source was last searched or consulted. | 4 |
| Search strategy | 7 | Present the full search strategies for all databases, registers and websites, including any filters and limits used. | 4 and Table S2 |
| Selection process | 8 | Specify the methods used to decide whether a study met the inclusion criteria of the review, including how many reviewers screened each record and each report retrieved, whether they worked independently, and if applicable, details of automation tools used in the process. | 4 |
| Data collection process | 9 | Specify the methods used to collect data from reports, including how many reviewers collected data from each report, whether they worked independently, any processes for obtaining or confirming data from study investigators, and if applicable, details of automation tools used in the process. | 4 |
| Data items | 10a | List and define all outcomes for which data were sought. Specify whether all results that were compatible with each outcome domain in each study were sought (e.g. for all measures, time points, analyses), and if not, the methods used to decide which results to collect. | 5 |
|  | 10b | List and define all other variables for which data were sought (e.g. participant and intervention characteristics, funding sources). Describe any assumptions made about any missing or unclear information. | 4,5 |
| Study risk of bias assessment | 11 | Specify the methods used to assess risk of bias in the included studies, including details of the tool(s) used, how many reviewers assessed each study and whether they worked independently, and if applicable, details of automation tools used in the process. | 5 |
| Effect measures | 12 | Specify for each outcome the effect measure(s) (e.g. risk ratio, mean difference) used in the synthesis or presentation of results. | 5 |
| Synthesis methods | 13a | Describe the processes used to decide which studies were eligible for each synthesis (e.g. tabulating the study intervention characteristics and comparing against the planned groups for each synthesis (item #5)). | 4,5 |
|  | 13b | Describe any methods required to prepare the data for presentation or synthesis, such as handling of missing summary statistics, or data conversions. | 4,5 |
|  | 13c | Describe any methods used to tabulate or visually display results of individual studies and syntheses. | 4,5 |
|  | 13d | Describe any methods used to synthesize results and provide a rationale for the choice(s). If meta-analysis was performed, describe the model(s), method(s) to identify the presence and extent of statistical heterogeneity, and software package(s) used. | 4,5 |
|  | 13e | Describe any methods used to explore possible causes of heterogeneity among study results (e.g. subgroup analysis, meta-regression). | NA |
|  | 13f | Describe any sensitivity analyses conducted to assess robustness of the synthesized results. | NA |
| Reporting bias assessment | 14 | Describe any methods used to assess risk of bias due to missing results in a synthesis (arising from reporting biases). | 5 |
| Certainty assessment | 15 | Describe any methods used to assess certainty (or confidence) in the body of evidence for an outcome. | 5 |
| **RESULTS** | | |  |
| Study selection | 16a | Describe the results of the search and selection process, from the number of records identified in the search to the number of studies included in the review, ideally using a flow diagram. | 5, Figure 1 |
|  | 16b | Cite studies that might appear to meet the inclusion criteria, but which were excluded, and explain why they were excluded. | NA |
| Study characteristics | 17 | Cite each included study and present its characteristics. | Table S3 |
| Risk of bias in studies | 18 | Present assessments of risk of bias for each included study. | 7-8 |
| Results of individual studies | 19 | For all outcomes, present, for each study: (a) summary statistics for each group (where appropriate) and (b) an effect estimate and its precision (e.g. confidence/credible interval), ideally using structured tables or plots. | Table 1-4, Page 8-9 |
| Results of syntheses | 20a | For each synthesis, briefly summarise the characteristics and risk of bias among contributing studies. | 7-8 |
|  | 20b | Present results of all statistical syntheses conducted. If meta-analysis was done, present for each the summary estimate and its precision (e.g. confidence/credible interval) and measures of statistical heterogeneity. If comparing groups, describe the direction of the effect. | Table 1-4, Page 8-9 |
|  | 20c | Present results of all investigations of possible causes of heterogeneity among study results. | NA |
|  | 20d | Present results of all sensitivity analyses conducted to assess the robustness of the synthesized results. | NA |
| Reporting biases | 21 | Present assessments of risk of bias due to missing results (arising from reporting biases) for each synthesis assessed. | NA |
| Certainty of evidence | 22 | Present assessments of certainty (or confidence) in the body of evidence for each outcome assessed. | NA |
| **DISCUSSION** | | |  |
| Discussion | 23a | Provide a general interpretation of the results in the context of other evidence. | 9 |
|  | 23b | Discuss any limitations of the evidence included in the review. | 11 |
|  | 23c | Discuss any limitations of the review processes used. | 11 |
|  | 23d | Discuss implications of the results for practice, policy, and future research. | 11 |
| **OTHER INFORMATION** | | |  |
| Registration and protocol | 24a | Provide registration information for the review, including register name and registration number, or state that the review was not registered. | NA |
|  | 24b | Indicate where the review protocol can be accessed, or state that a protocol was not prepared. | NA |
|  | 24c | Describe and explain any amendments to information provided at registration or in the protocol. | NA |
| Support | 25 | Describe sources of financial or non-financial support for the review, and the role of the funders or sponsors in the review. | 12 |
| Competing interests | 26 | Declare any competing interests of review authors. | 12 |
| Availability of data, code and other materials | 27 | Report which of the following are publicly available and where they can be found: template data collection forms; data extracted from included studies; data used for all analyses; analytic code; any other materials used in the review. | 12 |

NA: Not applicable.

**Table S2: Search strategy**

**PubMed**

(cervical dysplasia[Text Word] OR cervical intraepithelial neoplasia[Text Word] OR cervix neoplasms[Text Word] OR papillomavirus[Text Word] OR papillomavirus, human[Text Word] OR human papillomavirus[Text Word] OR papillomavirus, infections)AND (self-collected[Text Word] OR self-test[Text Word] OR self-obtained[Text Word] OR self-sampling[Text Word]) AND (Asia[Text Word] OR Asian[Text Word] OR Afghanistan[Text Word] OR Armenia[Text Word] OR Azerbaijan[Text Word] OR Bahrain[Text Word] OR Bangladesh[Text Word] OR Bhutan[Text Word] OR Brunei[Text Word] OR Cambodia[Text Word] OR China[Text Word] OR Cyprus[Text Word] OR Georgia[Text Word] OR India[Text Word] OR Indonesia[Text Word] OR Iran[Text Word] OR Iraq[Text Word] OR Israel[Text Word] OR Japan[Text Word] OR Jordan[Text Word] OR Kazakhstan[Text Word] OR Korea, North[Text Word] OR Korea, South[Text Word] OR Kuwait[Text Word] OR Kyrgyzstan[Text Word] OR Laos[Text Word] OR Lebanon[Text Word] OR Malaysia[Text Word] OR Maldives[Text Word] OR Mongolia[Text Word] OR Myanmar[Text Word] OR Nepal[Text Word] OR Oman[Text Word] OR Pakistan[Text Word] OR Palestine[Text Word] OR Philippines[Text Word] OR Qatar[Text Word] OR Saudi Arabia[Text Word] OR Singapore[Text Word] OR Sri Lanka[Text Word] OR Syria[Text Word] OR Tajikistan[Text Word] OR Thailand[Text Word] OR Timor-Leste[Text Word] OR Turkmenistan[Text Word] OR Turkey[Text Word] OR United Arab Emirates[Text Word] OR Uzbekistan[Text Word] OR Vietnam[Text Word] OR Yemen[Text Word]) AND ("1993/01/01"[Date - Publication] : "2023/02/23"[Date - Publication])

**Web of Science**

(TS=(cervical dysplasia OR cervical intraepithelial neoplasia OR cervix neoplasms OR papillomavirus OR papillomavirus, human OR human papillomavirus OR papillomavirus, infections)) AND (TS=(self-collected OR self-test OR self-obtained OR self-sampling)) AND (TS=(Asia OR Asian OR Afghanistan OR Armenia OR Azerbaijan OR Bahrain OR Bangladesh OR Bhutan OR Brunei OR Cambodia OR China OR Cyprus OR Georgia OR India OR Indonesia OR Iran OR Iraq OR Israel OR Japan OR Jordan OR Kazakhstan OR Korea, North OR Korea, South OR Kuwait OR Kyrgyzstan OR Laos OR Lebanon OR Malaysia OR Maldives OR Mongolia OR Myanmar OR Nepal OR Oman OR Pakistan OR Palestine OR Philippines OR Qatar OR Saudi Arabia OR Singapore OR Sri Lanka OR Syria OR Tajikistan OR Thailand OR Timor-Leste OR Turkmenistan OR Turkey OR United Arab Emirates OR Uzbekistan OR Vietnam OR Yemen)) AND (PY=(2001-2023))

**Cochrane library**

(cervical dysplasia OR cervical intraepithelial neoplasia OR cervix neoplasms OR papillomavirus OR papillomavirus, human OR human papillomavirus OR papillomavirus, infections) in All Text AND (self-collected OR self-test OR self-obtained OR self-sampling) in All Text AND (Asia OR Asian OR Afghanistan OR Armenia OR Azerbaijan OR Bahrain OR Bangladesh OR Bhutan OR Brunei OR Cambodia OR China OR Cyprus OR Georgia OR India OR Indonesia OR Iran OR Iraq OR Israel OR Japan OR Jordan OR Kazakhstan OR Korea, North OR Korea, South OR Kuwait OR Kyrgyzstan OR Laos OR Lebanon OR Malaysia OR Maldives OR Mongolia OR Myanmar OR Nepal OR Oman OR Pakistan OR Palestine OR Philippines OR Qatar OR Saudi Arabia OR Singapore OR Sri Lanka OR Syria OR Tajikistan OR Thailand OR Timor-Leste OR Turkmenistan OR Turkey OR United Arab Emirates OR Uzbekistan OR Vietnam OR Yemen) in All Text - (Word variations have been searched) AND Custom Range:1990-2023

**Cumulative Index to Nursing & Allied Health**

TX ( cervical dysplasia OR cervical intraepithelial neoplasia OR cervix neoplasms OR papillomavirus OR papillomavirus, human OR human papillomavirus OR papillomavirus, infections ) AND TX ( self-collected OR self-test OR self-obtained OR self-sampling ) AND TX ( Asia OR Asian OR Afghanistan OR Armenia OR Azerbaijan OR Bahrain OR Bangladesh OR Bhutan OR Brunei OR Cambodia OR China OR Cyprus OR Georgia OR India OR Indonesia OR Iran OR Iraq OR Israel OR Japan OR Jordan OR Kazakhstan OR Korea, North OR Korea, South OR Kuwait OR Kyrgyzstan OR Laos OR Lebanon OR Malaysia OR Maldives OR Mongolia OR Myanmar OR Nepal OR Oman OR Pakistan OR Palestine OR Philippines OR Qatar OR Saudi Arabia OR Singapore OR Sri Lanka OR Syria OR Tajikistan OR Thailand OR Timor-Leste OR Turkmenistan OR Turkey OR United Arab Emirates OR Uzbekistan OR Vietnam OR Yemen )

Limiters - Publication Date: 19900101-20230231; Language: English

Expanders - Apply equivalent subjects

Narrow by SubjectGender: - female

Search modes - Find all my search term

**Table S3: Characteristics of the studies included in the systematic review.**

**HPV= human papillomavirus; VIA= visual inspection with acetic acid; Pap= Papanicolaou cytology; HC2= hybrid capture II; PCR= polymerase chain reaction; HR-HPV= high-risk human papillomavirus; CIN= cervical intraepithelial neoplasia; ASCUS= atypical squamous cells of unknown significance.**

| **Author, Year** | **Country** | **Study Designs** | **Population Characteristics** | **Intervention** | **Clinical Outcomes** |
| --- | --- | --- | --- | --- | --- |
| Belinson, 2001[1] | China | Cross-sectional study | 1997 women aged 35–45 from rural Shanxi Province | Self-sampling, fluorescence spectroscopy, liquid-based Pap, VIA diagnosis, and colposcopy with multiple cervical biopsies | Accuracy of vaginal self-sampling |
| Chang, 2002[2] | China | Cross-sectional study | 1194 handicapped or elderly women living in rural area | HPV self-sampling and clinician sampling | Accuracy and concordance between HPV self-sampling and clinician sampling |
| Belinson, 2003[3] | China | Cross-sectional study | 8497 women aged 27—56 from rural Shanxi Province, China | Self-sampling versus clinician-sampling | Sensitivity and specificity of vaginal self-sampling |
| Tisci, 2003[4] | China | Cross-sectional study | 1560 women aged 35-50 years from Shanxi Province, and not had been screened for cervical neoplasia in the past 10 years | HPV self-sampling, interviewers' survey | Acceptance of cervicovaginal self-sampling for HPV and barriers associated with HPV self-sampling |
| Hanh, 2006[5] | Vietnam | Cross-sectional study | 1238 married women from northen Vietnam | HPV self-sampling, clinician sampling and questionnaires | Acceptability of self-sampling for cervical cancer screening and concordance between the self-sampling and physician-sampling methods |
| Seo, 2006[6] | Korea | Cross-sectional study | 118 women with with abnormal Pap smears | HPV self-sampling and clinician sampling | Accuracy and concordance between HPV self-sampling and clinician sampling |
| Bhatla, 2009[7] | India | Cross-sectional study | 546 sexually active women aged ≥30 years with persistent vaginal discharge, intermenstrual or postcoital bleeding or an unhealthy cervix | Self-sampling, clinician-sampling | Accuracy and concordance between HPV by HC2 and PCR on self-collected vaginal and physician-collected cervical samples and cytology |
| Sowjanya, 2009[8] | India | Cross-sectional study | 432 women aged from 25-85 years | Self-sampling, clinician-sampling | Detection rate of self-sampling and clinician-sampling, participation rates of self-sampling at home and clinic |
| Qiao, 2009[9] | China | Cross-sectional study | 2530 women aged 30-54 years in Shanxi province | HPV self-sampling and clinician sampling | Accuracy of HPV self-sampling and clinician sampling |
| Belinson, 2010[10] | China | Cross-sectional study | 2653 women aged 16-54 years from Henan, Xinjiang and Shanxi province | HPV self-collecting a vaginal specimen, physicians collected specimens from the perineum, lower and upper vagina and endocervix | Accuracy of HPV self-sampling and clinician sampling |
| Yoshida, 2011[11] | Japan | Cross-sectional study | 50 women aged 21–61 years with a prior diagnosis more than CIN1 | Self-sampling, clinician-sampling | Accuracy and concordance between HPV self-sampling and clinician sampling |
| Twu, 2011[12] | China | Cross-sectional study | 1717 women aged 26-79 years | Self-sampling, clinician-sampling | Detection and typing of HPV between vaginal and cervical specimens |
| Belinson, 2012[13] | China | Randomized controlled trial | 8556 women aged 25–59 years from Guangdong Province | Self-sampling, clinician-sampling | Sensitivity of self-collected specimens using different self-collection device and different test assays |
| Guan, 2012[14] | China | Cross-sectional study | 174 women from Shanxi Province | Self-sampling, clinician-sampling, colposcopy examination and questionnaires | Acceptability and preference of self-sampling |
| Guan, 2013[15] | China | Cross-sectional study | 174 women from Shanxi Province | Self-sampling, clinician-sampling | Agreement of self-versus clinician-collection specimens onto FTA cartridges for the detection of HPV |
| Trope, 2013[16] | Thailand | Cross-sectional study | 431 women aged 25-60 years, hadn't been screened for cervical cancer in the last 5 years in Roi-et Province | Self-sampling | Safety, acceptability and feasibility of primary HPV testing for cervical cancer prevention at the community level in a low-resource setting |
| Zhao, 2013[17] | China | Cross-sectional study | 7543 women aged 25-65 years and living in rural area | HPV self-sampling and clinician sampling | Accuracy of HPV self-sampling and clinician sampling |
| Johnson, 2014[18] | Nepal | Cross-sectional study | 261 women of reproductive age from Achham District of rural far western Nepal | Self-sampling, clinician-sampling | Distribution of among rural Nepali women and agreement between clinician- and self-collected tests |
| Chen, 2014[19] | China | Cross-sectional study | 500 women aged 18–65 years old from Taiwan province | Questionnaires | Determinants of women’s likelihood of HPV self-sampling |
| Oranratanaphan, 2014[20] | Thailand | Cross-sectional study | 100 women aged 30-65 years | Pap test by physicians, HPV self-sampling and questionnaires | Acceptability of self-Sampling HPV testing |
| Nilyanimit, 2014[21] | Thailand | Cross-sectional study | 101 females between ages 20-70 years from Bangkok | Physician-collected cervical swabs, self-collected vaginal swabs | The HPV DNA test results between self-collected and physician-collected cervical swabs |
| Chen Wen, 2014[22] | China | Cross-sectional study | 7500 women aged 25–65 years in rural area | A self-collected and two clinician-collected specimens and VIA | Agreement between careHPV and HC2 on self- and clinician-collected specimens. |
| Wang, 2014[23] | China | Cross-sectional study | 396 women aged 25-65 years | HPV self-sampling and clinician sampling | Accuracy of HPV self-sampling and clinician sampling |
| Asthana, 2015[24] | India | Cross-sectional study | 4658 ever-married women aged 30-59 years | HPV self-sampling and clinician sampling | Accuracy of HPV self-sampling and clinician sampling |
| Latiff, 2015[25] | Malaysia | Cross-sectional study | 486 women aged 20-71 years from rural District of Jempol, Negeri Sembilan | Self-sampling and clinician-sampling | Agreement and differences between self-sampling with a Kato device and gynecologist sampling for Pap cytology and HPV DNA detection |
| Latiff, 2015[26] | Malaysia | Cross-sectional study | 258 women in reproductive age (15-49 years old) from Selangor state | Self-sampling and physician-sampling | Reliability of self-sampling for cervical smear |
| Chou, 2016[27] | China | Prospective population-based study | 10693 women residing in Taoyuan City, and not having Pap smear screening in the past 5 years | Self-sampling and questionnaires | Feasibility of a self-sampling HPV test for under-users of Pap smear and factors associated with under-screening |
| Ma’som, 2016[28] | Malaysia | Cross-sectional study | 839 participants aged 18-60 years from Selangor | Self-sampling and questionnaires | Attitudes and acceptability of self-administered cervicovaginal sampling compared with Pap smear |
| Hanley, 2016[29] | Japan | Cross-sectional study | 203 women aged 20–49 attending their annual workplace health-check in Sapporo | Self-sampling, physician-sampling and questionnaires | Acceptability of self-Sampling HPV testing |
| Kittisiam, 2016[30] | Thailand | Cross-sectional study | 2810 women aged 25-65 years and lived in Bangkok for more than 5 years | Questionnaires | Knowledge and attitudes of Bangkok women regarding HPV and self-sampled HPV testing |
| Wong, 2016[31] | China | Prospective, randomized crossover study | 392 women aged 35-65 years in Hongkong | Self-sampling, pap smear and questionnaires | Acceptability of HPV DNA self-sampling and its impact on the rate of compliance with cervical cancer screening |
| Chen, 2016[32] | China | Cross-sectional study | 202 women consisted of 101 patients in Shanghai referred for abnormal cervical screening results and 101 women without cervical lesions | Self-sampling, physician-sampling and colposcopy examination with a Pap smear and directed or random biopsies | Analytical and clinical effectiveness of cervicovaginal self-sampling with a dry sampling device for testing and detection of cervical disease |
| Chen, Q 2016[33] | China | Cross-sectional study | 210 women aged 18-56 years with abnormal cervical cytology and presenting a positive HR-HPV test | HPV self-sampling and clinician sampling | Accuracy and concordance between HPV self-sampling and clinician sampling |
| Qin, 2016[34] | China | Cross-sectional study | 291 HIV-1 positive women aged 25-65 | HPV self-sampling and clinician sampling | Concordance between HPV self-sampling and clinician sampling |
| Aiko, 2017[35] | Japan | Cross-sectional study | 136 women aged 20–69 years with abnormal cervical cytology | Cervical samples for conventional cytology, physician-collected HPV testing, self-sampled vaginal specimen and questionnaires | Accuracy of self-collected vaginal samples compared with physician-collected cervical samples for testing |
|  |  |  |  |  |  |
| Wang, 2017[36] | China | Cross-sectional study | 2337 women aged 30-54 years in Shanxi province | HPV self-sampling and clinician sampling | Accuracy and concordance between HPV self-sampling and clinician sampling |
| Wong, 2018[37] | China | Cross-sectional study | 68 female sex workers, aged 22-59 years | HPV DNA self-sampling, clinician sampling and questionnaires | Acceptability and reliability of HPV DNA self-sampling |
| Abdullah, 2018[38] | Malaysia | Cross-sectional study | 164 women aged 28–60 years | HPV self-sampling and questionnaires | Acceptability of HPV self-sampling |
| Phoolcharoen, 2018[39] | Thailand | Cross-sectional study | 247 women aged 30–70 years from Bangkok | HPV DNA self-sampling, clinician-sampling and questionnaires | Acceptability of the self-sample HPV screening method |
| Phoolcharoen, 2018[40] | Thailand | Cross-sectional study | 247 women aged 30–70 years who visited a colposcopy clinic at Bangkok | HPV DNA self-sampling and clinician-sampling | Concordance between vaginal self- and endocervical physician-collected testing |
| Thay, 2019[41] | Cambodia | Prospective cohort study | 250 Cambodian women (129 HIV+, 121 HIV-) | HPV self-sampling, clinician sampling, VIA, digital colposcopy | Sensitivity of self-sampled HPV testing as compared with clinician-collected samples |
| Nutthachot, 2019[42] | Thailand | Cross-sectional study | 400 women attending for routine cervical cancer screening | HPV self-sampling and clinician sampling | Correlation between self-collected and clinician-collected HPV DNA testing |
|  |  |  |  |  |  |
| Cho, 2019[43] | Korea | Cross-sectional study | 101 women aged 20-50 years | HPV self-sampling and clinician sampling | Concordance between HPV self-sampling and clinician sampling with different assays |
| Gottschlich, 2019[44] | Thailand | Cross-sectional study | 267 women from the Buddhist district of Ranot and Muslim district of Na Thawi, Songkhla | HPV self-sampling and questionnaire | Acceptability of self-sampling for cervical cancer screening |
| Shin, 2019[45] | Korea | Cross-sectional study | 732 women aged between 20 and 69 years | HPV DNA self-sampling, clinician sampling and questionnaire | Acceptability of self-sampling for cervical cancer screening |
| Khoo, 2020[46] | Malaysia | Cross-sectional study | 725 women aged 35 to 45 years from the urban and suburban areas of Selangor | HPV self-sampling and questionnaires | Acceptability of vaginal self-sampling for HPV testing |
| Onuma, 2020[47] | Japan | Cross-sectional study | 100 patients who had previously tested negative for intraepithelial lesions or malignancy/HPV-positive, and patients with ASCUS or worse cytology | HPV self-sampling, clinician sampling, colposcopy and biopsy | Concordance in HPV type between self- and physician-collected samples |
| Satake, 2020[48] | Japan | Cross-sectional study | 300 women aged 20-59 years who visited 3 private hospitals in Sapporo city | HPV self-sampling and clinician sampling | Overall concordance rate of test and cytology using self-sampled and physician-sampled cervicovaginal tests |
| Wong, 2020[49] | China | Cross-sectional study | 177 women aged 25–35 and aged ≥45 in Hongkong | HPV self-sampling and questionnaire | Acceptability and feasibility of HPV self-sampling |
| Kuriakose, 2020[50] | India | Cross-sectional study | 114 patients with newly diagnosed cervical cancer | HPV DNA self-sampling and clinician-sampling | Accuracy of self-collected vaginal samples for HPV DNA detection |
| Goldstein, 2020[51] | China | Cross-sectional study | 600 women aged 35–65 years in the Yunnan province | HPV self-sampling and questionnaire | Patient satisfaction with HPV self-Sampling |
| He, 2020[52] | China | Cross-sectional study | 1810 healthy women undergoing routine physical examinations at the West China Hospital, Sichuan University | Questionnaires | Attitudes towards HPV self-sampling |
| Zhang, 2020[53] | China | Cross-sectional study | 6042 women ged 30-55 years who had not been screened for cervical cancer in the 3 years | HPV self-sampling and clinician sampling | Accuracy of HPV self-sampling and clinician sampling |
| Tan, 2021[54] | Malaysia | Cross-sectional study | 55 women aged between 20-80 years were recruited via convenient sampling from villages in Long Banga, Sarawak | HPV self-sampling and questionnaires | Feasibility of providing primary HPV DNA test using the self- sampling method to the hard-to-reach population in the interior of Sarawak during the COVID-19 pandemic |
| Madhivanan, 2021[55] | India | Cross-sectional study | 120 women over the age of 30 in rural villages in Mysore District | HPV DNA self-sampling, clinician sampling and questionnaires | Acceptability and concordance of self-vs. clinician-collected samples for HPV related cervical cancer screening |
| Du, 2021[56] | China | Cross-sectional study | 10339 women aged 30-59 years | HPV self-sampling and clinician sampling | Accuracy of HPV self-sampling and clinician sampling |
| Ahmad, 2021[57] | Malaysia | Cross-sectional study | 220 women aged between 18 to 55 years | Questionnaire | Acceptability of self-sampling for cervical cancer screening |
| Shrestha, 2021[58] | Nepal | Cross-sectional study | 30 healthy women | Semi-structured in-depth interviews | Acceptability of self-sampling for cervical cancer screening |
| Tsedenbal, 2022[59] | Mongolia | Cross-sectional study | 175 women who wished to be screened for cervical cancer | HPV self-sampling and questionnaires | Acceptability of physician-sampling and self-sampling |
| Chaw, 2022[60] | Brunei | Cross-sectional study | 174 women aged between 20 and 65 years old and had never undergone cervical cancer screening, or did not have one in 3 years | HPV self-sampling and questionnaires | Reasons for non-attendance and explore their acceptance of HPV self-sampling |
| Ngu, 2022[61] | China | Cross-sectional study | 521 women aged 30–65 years who were never-or under-screened | HPV self-sampling, clinician sampling and questionnaires | Effectiveness of HPV self-sampling for cervical cancer screening and the best means of service delivery, with a specific focus on under-screened women, particularly during the COVID-19 pandemic |
| Lim, 2022[62] | Singapore | Prospective, randomized crossover study | 300 women aged 30–69 years | HPV self-sampling, clinician sampling and questionnaires | Acceptability of self-sampling for cervical cancer screening and concordance between the self-sampling and physician-sampling methods |
| Anand, 2022[63] | India | Cross-sectional study | 1000 women aged 30-55 years | HPV self-sampling and clinician sampling | Concordance between HPV self-sampling and clinician sampling, acceptability of self-sampling for cervical cancer screening |
| Li, 2022[64] | China | Cross-sectional study | 20103 women aged 30–59 years and participated registered on a cervical cancer screening website | HPV self-sampling and questionnaire | Acceptability of HPV self-sampling combined with the internet |
| Terada, 2022[65] | Japan | Cross-sectional study | 300 women aged 20-50 years with abnormal cervical cytology | HPV self-sampling and clinician sampling | Concordance between HPV self-sampling and clinician sampling |
| Ozawa, 2023[66] | Japan | Cross-sectional study | 165 women aged 20–50 years | HPV self-sampling device–sponge type, physician-sampling HPV testing and cytology and questionnaires | Feasibility of human papillomavirus sponge-type self-sampling device |
| Ploysawang, 2023[67] | Thailand | Cross-sectional study | 265 women aged 30-60 years | HPV self-sampling, clinician sampling and questionnaire | Acceptability of HPV self-sampling combined with the internet |

**Reference**

1. Belinson J, Qiao YL, Pretorius R, et al. Shanxi Province Cervical Cancer Screening Study: a cross-sectional comparative trial of multiple techniques to detect cervical neoplasia. Gynecol Oncol. 2001 Nov;83(2):439–44. doi: 10.1006/gyno.2001.6370.

2. Chang CC, Tseng CJ, Liu W wei,et al. Clinical evaluation of a new model of self-obtained method for the assessment of genital human papilloma virus infection in an underserved population. Chang Gung Med J. 2002 Oct;25(10):664–71.

3. Belinson JL, Qiao YL, Pretorius RG, et al. Shanxi Province cervical cancer screening study II: self-sampling for high-risk human papillomavirus compared to direct sampling for human papillomavirus and liquid based cervical cytology. Int J Gynecol Cancer Off J Int Gynecol Cancer Soc. 2003;13(6):819–26. doi: 10.1111/j.1525-1438.2003.13611.x.

4. Tisci S, Shen YH, Fife D, et al. Patient acceptance of self-sampling for human papillomavirus in rural china. J Low Genit Tract Dis. 2003 Apr;7(2):107–16. doi: 10.1097/00128360-200304000-00007.

5. Hanh La. Epidemiology of high-risk HPV infection in Northern Vietnam among married women and tolerability of self testing. United States -- Maryland: The Johns Hopkins University; 2006. Accession Number: PQDT:56287088.

6. Seo SS, Song YS, Kim JW, et al. Good correlation of HPV DNA test between self-collected vaginal and clinician-collected cervical samples by the oligonucleotide microarray. Gynecol Oncol. 2006 Jul;102(1):67–73. doi: 10.1016/j.ygyno.2005.11.030.

7. Bhatla N, Dar L, Patro AR, et al. Can human papillomavirus DNA testing of self-collected vaginal samples compare with physician-collected cervical samples and cytology for cervical cancer screening in developing countries? Cancer Epidemiol. 2009 Dec;33(6):446–50. doi: 10.1016/j.canep.2009.10.013.

8. Sowjanya AP, Paul P, Vedantham H, et al. Suitability of self-collected vaginal samples for cervical cancer screening in periurban villages in Andhra Pradesh, India. Cancer Epidemiol Biomark Prev Publ Am Assoc Cancer Res Cosponsored Am Soc Prev Oncol. 2009 May;18(5):1373–8. doi: 10.1158/1055-9965.EPI-08-1171.

9. Qiao YL, Sellors JW, Eder PS, et al. A new HPV-DNA test for cervical-cancer screening in developing regions: a cross-sectional study of clinical accuracy in rural China. Lancet Oncol. 2008 Oct;9(10):929–36. doi: 10.1016/S1470-2045(08)70210-9.

1. Belinson JL, Hu S, Niyazi M, et al. Prevalence of type-specific human papillomavirus in endocervical, upper and lower vaginal, perineal and vaginal self-collected specimens: Implications for vaginal self-collection. Int J Cancer. 2010 Sep 1;127(5):1151–7. doi: 10.1002/ijc.25144.
2. Yoshida T, Sano T, Takada N, et al. Comparison of self-collected and clinician-collected materials for cervical cytology and human papillomavirus genotyping: analysis by linear array assay. Acta Cytol. 2011;55(1):106–12. doi: 10.1159/000320924.
3. Twu NF, Yen MS, Lau HY, et al. Type-specific human papillomavirus DNA testing with the genotyping array: a comparison of cervical and vaginal sampling. Eur J Obstet Gynecol Reprod Biol. 2011 May;156(1):96–100. doi: 10.1016/j.ejogrb.2010.12.023.
4. Belinson JL, Du H, Yang B, et al. Improved sensitivity of vaginal self-collection and high-risk human papillomavirus testing. Int J Cancer. 2012 Apr 15;130(8):1855–60. doi: 10.1002/ijc.26202.
5. Guan Y, Castle PE, Wang S, et al. A cross-sectional study on the acceptability of self-collection for HPV testing among women in rural China. Sex Transm Infect. 2012 Nov;88(7):490–4. doi: 10.1136/sextrans-2012-050477.
6. Guan Y, Gravitt PE, Howard R, et al. Agreement for HPV genotyping detection between self-collected specimens on a FTA cartridge and clinician-collected specimens. J Virol Methods. 2013 Apr;189(1):167. doi: 10.1016/j.jviromet.2012.11.010.
7. Trope LA, Chumworathayi B, Blumenthal PD. Feasibility of community-based careHPV for cervical cancer prevention in rural Thailand. J Low Genit Tract Dis. 2013 Jul;17(3):315–9. doi: 10.1097/LGT.0b013e31826b7b70.
8. Zhao FH, Jeronimo J, Qiao YL, et al. An evaluation of novel, lower-cost molecular screening tests for human papillomavirus in rural China. Cancer Prev Res (Phila). 2013;6(9):938-948. doi:10.1158/1940-6207.CAPR-13-0091
9. Johnson DC, Bhatta MP, Smith JS, et al. Assessment of high-risk human papillomavirus infections using clinician- and self-collected cervical sampling methods in rural women from far western Nepal. PloS One. 2014;9(6):e101255. doi: 10.1371/journal.pone.0101255.
10. Chen SL, Hsieh PC, Chou CH, et al. Determinants of women’s likelihood of vaginal self-sampling for human papillomavirus to screen for cervical cancer in Taiwan: a cross-sectional study. BMC Womens Health. 2014 Nov 25;14:139. doi: 10.1186/s12905-014-0139-0.
11. Oranratanaphan S, Termrungruanglert W, Khemapech N. Acceptability of self-sampling HPV testing among Thai women for cervical cancer screening. Asian Pac J Cancer Prev APJCP. 2014;15(17):7437–41. doi: 10.7314/apjcp.2014.15.17.7437.
12. Nilyanimit P. Comparison of detection sensitivity for human papillomavirus between self-collected vaginal swabs and physician-collected cervical swabs by electrochemical DNA chip. Asian Pac J Cancer Prev APJCP. 2014;15(24):10809–12.
13. Chen W, Jeronimo J, Zhao FH, et al. The concordance of HPV DNA detection by Hybrid Capture 2 and careHPV on clinician- and self-collected specimens. J Clin Virol Off Publ Pan Am Soc Clin Virol. 2014 Dec;61(4):553–7. doi: 10.1016/j.jcv.2014.09.018.
14. Wang SM, Hu SY, Chen F, et al. Clinical evaluation of human papillomavirus detection by careHPV™ test on physician-samples and self-samples using the indicating FTA Elute® card. Asian Pac J Cancer Prev. 2014;15(17):7085-7089. doi:10.7314/apjcp.2014.15.17.7085.
15. Asthana S, Labani S. Adjunct screening of cervical or vaginal samples using careHPV testing with Pap and aided visual inspection for detecting high-grade cervical intraepithelial neoplasia. Cancer Epidemiol. 2015 Feb 1;39(1):104–8. oi: 10.1016/j.canep.2014.11.006.
16. Lattiff (2) 2015: Latiff LA, Ibrahim Z, Pei CP, et al. Comparative Assessment of a Self-sampling Device and Gynecologist Sampling for Cytology and HPV DNA Detection in a Rural and Low Resource Setting: Malaysian Experience. Asian Pac J Cancer Prev. 2015;16(18):8495-8501. doi:10.7314/apjcp.2015.16.18.8495.
17. Lattiff 2015: Latiff LA, Rahman SA, Wee WY, et al. Assessment of the reliability of a novel self-sampling device for performing cervical sampling in Malaysia. Asian Pac J Cancer Prev. 2015;16(2):559-564. doi:10.7314/apjcp.2015.16.2.559.
18. Chou HH, Huang HJ, Cheng HH, et al. Self-sampling HPV test in women not undergoing Pap smear for more than 5 years and factors associated with under-screening in Taiwan. J Formos Med Assoc Taiwan Yi Zhi. 2016 Dec;115(12):1089–96. doi: 10.1016/j.jfma.2015.10.014.
19. Ma’som M, Bhoo-Pathy N, Nasir NH, et al. Attitudes and factors affecting acceptability of self-administered cervicovaginal sampling for human papillomavirus (HPV) genotyping as an alternative to Pap testing among multiethnic Malaysian women. BMJ Open. 2016 Aug 4;6(8):e011022. doi: 10.1136/bmjopen-2015-011022.
20. Hanley SJ, Fujita H, Yokoyama S, et al. HPV self-sampling in Japanese women: A feasibility study in a population with limited experience of tampon use. J Med Screen. 2016 Sep;23(3):164–70. doi: 10.1177/0969141315625702.
21. Kittisiam T, Tangjitgamol S, Chaowawanit W, et al. Knowledge and Attitudes of Bangkok Metropolitan Women towards HPV and Self-Sampled HPV Testing. Asian Pac J Cancer Prev APJCP. 2016;17(5):2445–51.
22. Wong ELY, Chan PKS, Chor JSY, et al. Evaluation of the Impact of Human Papillomavirus DNA Self-sampling on the Uptake of Cervical Cancer Screening. Cancer Nurs. 2016;39(1):E1–11. doi: 10.1097/NCC.0000000000000241.
23. Chen K, Ouyang Y, Hillemanns P, et al. Excellent analytical and clinical performance of a dry self-sampling device for human papillomavirus detection in an urban Chinese referral population. J Obstet Gynaecol Res. 2016 Dec;42(12):1839–45.doi: 10.1111/jog.13132.
24. Chen Q, Du H, Zhang R, et al. Evaluation of novel assays for the detection of human papilloma virus in self-collected samples for cervical cancer screening. Genet Mol Res. 2016;15(2):10.4238/gmr.15027896. doi:10.4238/gmr.15027896.
25. Qin Y, Zhang H, Marlowe N, et al. Evaluation of human papillomavirus detection by Abbott m2000 system on samples collected by FTA EluteTM Card in a Chinese HIV-1 positive population. J Clin Virol Off Publ Pan Am Soc Clin Virol. 2016 Dec;85:80–5. doi: 10.1016/j.jcv.2016.11.002.
26. Aiko K, Yoko M, Saito OM, et al. Accuracy of self‐collected human papillomavirus samples from Japanese women with abnormal cervical cytology. J Obstet Gynaecol Res. 2017 Apr;43(4):710–7. doi: 10.1111/jog.13258.
27. Wang M, Hu S, Zhao S, et al. Accuracy of triage strategies for human papillomavirus DNA-positive women in low-resource settings: A cross-sectional study in China. Chin J Cancer Res Chung-Kuo Yen Cheng Yen Chiu. 2017 Dec;29(6):496–509. doi: 10.21147/j.issn.1000-9604.2017.06.04.
28. Wong ELY, Cheung AWL, Huang F, et al. Can Human Papillomavirus DNA Self-sampling be an Acceptable and Reliable Option for Cervical Cancer Screening in Female Sex Workers? Cancer Nurs. 2018;41(1):45–52. doi: 10.1097/NCC.0000000000000462.
29. Abdullah NN, Daud S, Wang SM, et al. Human Papilloma Virus (HPV) self-sampling: do women accept it? J Obstet Gynaecol. 2018 Apr 3;38(3):402–7. doi: 10.1080/01443615.2017.1379061.
30. Phoolcharoen N, Kantathavorn N, Krisorakun W, et al. Acceptability of Self-Sample Human Papillomavirus Testing Among Thai Women Visiting a Colposcopy Clinic. J Community Health. 2018 Jun;43(3):611–5. doi: 10.1007/s10900-017-0460-2.
31. Phoolcharoen N, Kantathavorn N, Krisorakun W, et al. Agreement of self- and physician-collected samples for detection of high-risk human papillomavirus infections in women attending a colposcopy clinic in Thailand. BMC Res Notes. 2018 Feb 20;11(1):136. doi: 10.1186/s13104-018-3241-9.
32. Thay S, Goldstein A, Goldstein LS, et al. Prospective cohort study examining cervical cancer screening methods in HIV-positive and HIV-negative Cambodian Women: a comparison of human papilloma virus testing, visualization with acetic acid and digital colposcopy. BMJ Open. 2019 Feb 24;9(2):e026887. doi: 10.1136/bmjopen-2018-026887.
33. Nutthachote P, Oranratanaphan S, Termrungruanglert W, et al. Comparison of detection rate of high risk HPV infection between self-collected HPV testing and clinician-collected HPV testing in cervical cancer screening. Taiwan J Obstet Gynecol. 2019 Jul;58(4):477–81. doi: 10.1016/j.tjog.2019.05.008.
34. Cho HW, Ouh YT, Hong JH, et al. Comparison of urine, self-collected vaginal swab, and cervical swab samples for detecting human papillomavirus (HPV) with Roche Cobas HPV, Anyplex II HPV, and RealTime HR-S HPV assay. J Virol Methods. 2019 Jul;269:77–82. doi: 10.1016/j.jviromet.2019.04.012.
35. Gottschlich A, Nuntadusit T, Zarins KR, et al. Barriers to cervical cancer screening and acceptability of HPV self-testing: a cross-sectional comparison between ethnic groups in Southern Thailand. BMJ Open. 2019 Nov 3;9(11):e031957. doi: 10.1136/bmjopen-2019-031957.
36. Shin HY, Lee B, Hwang SH, et al. Evaluation of satisfaction with three different cervical cancer screening modalities: clinician-collected Pap test vs. HPV test by self-sampling vs. HPV test by urine sampling. J Gynecol Oncol. 2019 Sep;30(5):e76. doi: 10.3802/jgo.2019.30.e76.
37. Khoo SP, Lim WT, Rajasuriar R, et al. The Acceptability and Preference of Vaginal Self-sampling for Human Papillomavirus (HPV) Testing among a Multi-ethnic Asian Female Population. Cancer Prev Res Phila Pa. 2021 Jan;14(1):105–12. doi: 10.1158/1940-6207.CAPR-20-0280.
38. Onuma T, Kurokawa T, Shinagawa A, et al. Evaluation of the concordance in HPV type between self- and physician-collected samples using a brush-based device and a PCR-based HPV DNA test in Japanese referred patients with abnormal cytology or HPV infection. Int J Clin Oncol. 2020;25(10):1854-1860. doi:10.1007/s10147-020-01727-5.
39. Satake H, Inaba N, Kanno K, et al. Comparison Study of Self-Sampled and Physician-Sampled Specimens for High-Risk Human Papillomavirus Test and Cytology. Acta Cytol. 2020;64(5):433-441. doi:10.1159/000507342.
40. Wong ELY, Cheung AWL, Wong AYK, et al. Acceptability and Feasibility of HPV Self-Sampling as an Alternative Primary Cervical Cancer Screening in Under-Screened Population Groups: A Cross-Sectional Study. Int J Environ Res Public Health. 2020 Aug 27;17(17):6245. doi: 10.3390/ijerph17176245.
41. Kuriakose S, Sabeena S, Binesh D, et al. Diagnostic accuracy of self-collected vaginal samples for HPV DNA detection in women from South India. Int J Gynaecol Obstet Off Organ Int Fed Gynaecol Obstet. 2020 May;149(2):219–24. doi: 10.1002/ijgo.13116.
42. Goldstein A, Goldstein LS, Lipson R, et al. Assessing the feasibility of a rapid, high-volume cervical cancer screening programme using HPV self-sampling and digital colposcopy in rural regions of Yunnan, China. BMJ Open. 2020 Mar 30;10(3):e035153. doi: 10.1136/bmjopen-2019-035153.
43. He L, He J. Attitudes towards HPV self-sampling among women in Chengdu, China: A cross-sectional survey. J Med Screen. 2020 Dec;27(4):201–6. doi: 10.1177/0969141319895543.
44. Zhang W, Du H, Huang X, et al. Evaluation of an isothermal amplification HPV detection assay for primary cervical cancer screening. Infect Agent Cancer. 2020;15:65. doi: 10.1186/s13027-020-00328-1.
45. Taneepanichskul S, Lertmaharit S, Pongpanich S, et al. Quality of life among Thai women diagnosed with cervical cancer and cervical intraepithelial neoplasia at King Chulalongkorn Memorial Hospital. J Med Assoc Thail Chotmaihet Thangphaet. 2011 Aug;94(8):902–7.
46. Madhivanan P, Nishimura H, Ravi K, et al. Acceptability and Concordance of Self- Versus Clinician- Sampling for HPV Testing among Rural South Indian Women. Asian Pac J Cancer Prev APJCP. 2021 Mar 1;22(3):971–6. doi: 10.31557/APJCP.2021.22.3.971.
47. Du H, Luo H, Wang C, et al. The prevalence of HR-HPV infection based on self-sampling among women in China exhibited some unique epidemiologic features. J Clin Epidemiol. 2021 Nov;139:319–29. doi: 10.1016/j.jclinepi.2021.06.009.
48. Ahmad ZA, Daud S, Abdullah NN, et al. Perception and Knowledge of Human Papillomavirus (hpv) and Hpv Dna Self-Sampling Amongst Women in West Malaysia. Brunei Int Med J BIMJ. 2021 Jan;17:79–85.
49. Shrestha S, Thapa S, Sims P, et al. Feasibility of HPV self-sampling pathway in Kathmandu Valley, Nepal using a human-centred design approach. Sex Reprod Health Matters. 2021;29(3):2005283. doi: 10.1080/26410397.2021.2005283.
50. Tsedenbal B, Enebish G, Tserensodnom B, et al. Results of Self-Sampling Methodology Impression for Cervical Cancer Screening in Mongolia. Asian Pac J Cancer Prev APJCP. 2022 Dec 1;23(12):4099–107. doi: 10.31557/APJCP.2022.23.12.4099.
51. Chaw L, Lee SHF, Ja’afar NIH, et al. Reasons for non-attendance to cervical cancer screening and acceptability of HPV self-sampling among Bruneian women: A cross-sectional study. PloS One. 2022;17(3):e0262213. doi: 10.1371/journal.pone.0262213.
52. Ngu SF, Lau LSK, Li J, et al. Human Papillomavirus Self-Sampling for Primary Cervical Cancer Screening in Under-Screened Women in Hong Kong during the COVID-19 Pandemic. Int J Environ Res Public Health. 2022 Feb 24;19(5):2610. doi: 10.3390/ijerph19052610.
53. Lim LM, Chan MFG, Win PPT, et al. Self-sampling HPV DNA test for cervical cancer screening in Singapore: A prospective study. Ann Acad Med Singapore. 2022 Nov;51(11):733–5. doi: 10.47102/annals-acadmedsg.2022133.
54. Anand KV, Mishra GA, Pimple SA, et al. Cross-Sectional Study of HPV Self-Sampling among Indian Women—A Way Forward. Indian J Med Paediatr Oncol. 2022 Feb;43(01):103–8.
55. Li J, Wu R, Qu X, et al. Effectiveness and feasibility of self-sampling for human papillomavirus testing for internet-based cervical cancer screening. Front Public Health. 2022;10:938272. doi: 10.3389/fpubh.2022.938272.
56. Terada N, Matsuura M, Kurokawa S, et al. Human papillomavirus testing and cytology using physician-collected uterine cervical samples vs. self-collected vaginal samples and urine samples. Int J Clin Oncol. 2022;27(11):1742-1749. doi:10.1007/s10147-022-02238-1.
57. Ozawa N, Kurokawa T, Hareyama H, et al. Evaluation of the feasibility of human papillomavirus sponge-type self-sampling device at Japanese colposcopy clinics. J Obstet Gynaecol Res. 2023 Feb;49(2):701–8. doi: 10.1111/jog.15496.
58. Ploysawang P, Pitakkarnkul S, Kolaka W, et al. Acceptability and Preference for Human Papilloma Virus Self-Sampling among Thai Women Attending National Cancer Institute. Asian Pac J Cancer Prev. 2023;24(2):607-612. doi:10.31557/APJCP.2023.24.2.607.
